# Integrated multi-modal brain signatures predict sex-specific obesity status

**DOI:** 10.1093/braincomms/fcad098

**Published:** 2023-04-04

**Authors:** Ravi R Bhatt, Svetoslav Todorov, Riya Sood, Soumya Ravichandran, Lisa A Kilpatrick, Newton Peng, Cathy Liu, Priten P Vora, Neda Jahanshad, Arpana Gupta

**Affiliations:** Imaging Genetics Center, Mark and Mary Stevens Neuroimaging and Informatics Institute, University of Southern California, Marina del Rey, CA, 90089, USA; Goodman-Luskin Microbiome Center, G. Oppenheimer Center for Neurobiology of Stress and Resilience, Vatche and Tamar Manoukian Division of Digestive Diseases, Ingestive Behavior and Obesity Program, David Geffen School of Medicine at UCLA, Los Angeles, CA, 90095, USA; Goodman-Luskin Microbiome Center, G. Oppenheimer Center for Neurobiology of Stress and Resilience, Vatche and Tamar Manoukian Division of Digestive Diseases, Ingestive Behavior and Obesity Program, David Geffen School of Medicine at UCLA, Los Angeles, CA, 90095, USA; Goodman-Luskin Microbiome Center, G. Oppenheimer Center for Neurobiology of Stress and Resilience, Vatche and Tamar Manoukian Division of Digestive Diseases, Ingestive Behavior and Obesity Program, David Geffen School of Medicine at UCLA, Los Angeles, CA, 90095, USA; Goodman-Luskin Microbiome Center, G. Oppenheimer Center for Neurobiology of Stress and Resilience, Vatche and Tamar Manoukian Division of Digestive Diseases, Ingestive Behavior and Obesity Program, David Geffen School of Medicine at UCLA, Los Angeles, CA, 90095, USA; Goodman-Luskin Microbiome Center, G. Oppenheimer Center for Neurobiology of Stress and Resilience, Vatche and Tamar Manoukian Division of Digestive Diseases, Ingestive Behavior and Obesity Program, David Geffen School of Medicine at UCLA, Los Angeles, CA, 90095, USA; Goodman-Luskin Microbiome Center, G. Oppenheimer Center for Neurobiology of Stress and Resilience, Vatche and Tamar Manoukian Division of Digestive Diseases, Ingestive Behavior and Obesity Program, David Geffen School of Medicine at UCLA, Los Angeles, CA, 90095, USA; Goodman-Luskin Microbiome Center, G. Oppenheimer Center for Neurobiology of Stress and Resilience, Vatche and Tamar Manoukian Division of Digestive Diseases, Ingestive Behavior and Obesity Program, David Geffen School of Medicine at UCLA, Los Angeles, CA, 90095, USA; Imaging Genetics Center, Mark and Mary Stevens Neuroimaging and Informatics Institute, University of Southern California, Marina del Rey, CA, 90089, USA; Goodman-Luskin Microbiome Center, G. Oppenheimer Center for Neurobiology of Stress and Resilience, Vatche and Tamar Manoukian Division of Digestive Diseases, Ingestive Behavior and Obesity Program, David Geffen School of Medicine at UCLA, Los Angeles, CA, 90095, USA

**Keywords:** obesity, multi-modal brain imaging, machine learning, data integration, sex differences

## Abstract

Investigating sex as a biological variable is key to determine obesity manifestation and treatment response. Individual neuroimaging modalities have uncovered mechanisms related to obesity and altered ingestive behaviours. However, few, if any, studies have integrated data from multi-modal brain imaging to predict sex-specific brain signatures related to obesity. We used a data-driven approach to investigate how multi-modal MRI and clinical features predict a sex-specific signature of participants with high body mass index (overweight/obese) compared to non-obese body mass index in a sex-specific manner. A total of 78 high body mass index (55 female) and 105 non-obese body mass index (63 female) participants were enrolled in a cross-sectional study. All participants classified as high body mass index had a body mass index greater than 25 kg/m^2^ and non-obese body mass index had a body mass index between 19 and 20 kg/m^2^. Multi-modal neuroimaging (morphometry, functional resting-state MRI and diffusion-weighted scan), along with a battery of behavioural and clinical questionnaires were acquired, including measures of mood, early life adversity and altered ingestive behaviours. A Data Integration Analysis for Biomarker discovery using Latent Components was conducted to determine whether clinical features, brain morphometry, functional connectivity and anatomical connectivity could accurately differentiate participants stratified by obesity and sex. The derived models differentiated high body mass index against non-obese body mass index participants, and males with high body mass index against females with high body mass index obtaining balanced accuracies of 77 and 75%, respectively. Sex-specific differences within the cortico-basal-ganglia-thalamic-cortico loop, the choroid plexus-CSF system, salience, sensorimotor and default-mode networks were identified, and were associated with early life adversity, mental health quality and greater somatosensation. Results showed multi-modal brain signatures suggesting sex-specific cortical mechanisms underlying obesity, which fosters clinical implications for tailored obesity interventions based on sex.

## Teaser

Bhatt *et al*. report using multi-modal MRI and behavioural measures, multi-omics integration can predict sex-specfic obesity status. Obese individuals showed lower integrity of the cortico-basal-ganglia-thalamo-cortico loop and choroid plexus-CSF system alterations associated with early life trauma. Obese females show lower amygdala–sensorimotor connectivity and salience network integrity associated with greater somatization.

## Graphical Abstract

**Graphical Abstract:**
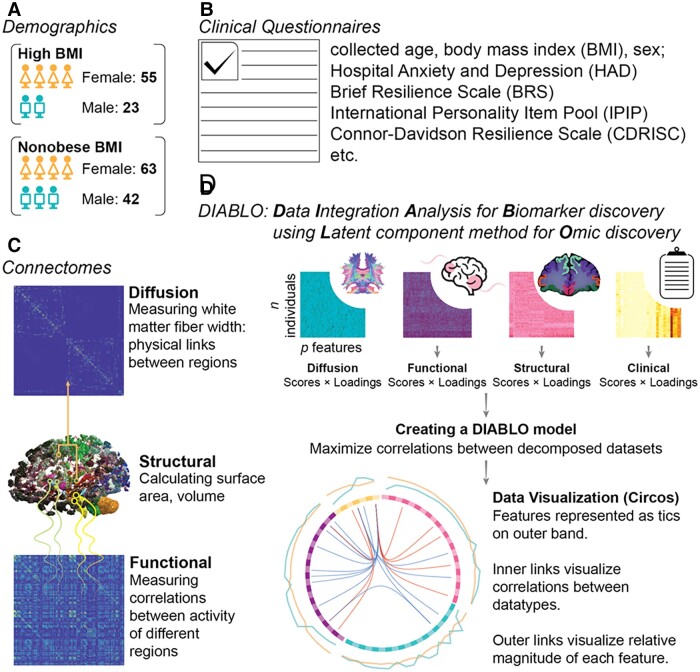

## Introduction

With over 42% of adults obese in the United States alone, the obesity epidemic constitutes a major global threat, with healthcare costs of over 700 billion dollars annually.^1–3^

Research depicting sex differences in obesity have recently gained momentum in an effort to improve the efficacy of current treatments, which at present are sub-optimal and do not have sustained outcomes.^4^ Females with obesity report nearly double the rates of food addiction compared to males (12.2 versus 6.4%, respectively),^5–7^ and increased food cravings and heightened reactivity to food cues, leading to a greater incidence of hedonic eating behaviours.^7,8^

Multi-modal MRI has helped understand the underlying obesity-related differences in brain structure and function.^9^ Compared to healthy controls, individuals with obesity show a heightened activation in reward and salience networks, including brains regions involved in memory functions, in response to visual food cues.^10^ This shown association with an increased appetite and greater food consumption, could explain the prevalence of overeating in individuals with obesity.^11^ Studies have identified the reward network as important to the pathophysiology of obesity due to its function in controlling voluntary behaviour.^12^ For example, altered connectivity in the basal ganglia may be attributed to a reduction in dopamine (D2) receptors, which modulate reward circuitry and emotional processing.^10,13^ The diminished sensitivity of these receptors alter intrinsic properties of stimuli, such as food palatability, and induce chronic overeating behaviours as a compensatory mechanism that develops into obesity pathology.^14,15^

Sex differences have also been observed in individuals with obesity regarding attention, memory and impulsivity domains.^16^ An inability to handle large cognitive loads leads to lowered cognitive restraint and higher anxiety, causing uncontrolled eating behaviours, which have a greater incidence in females than in males.^17,18^ Furthermore, a few studies have illustrated greater connectivity in emotional regulation regions in females with obesity is associated with their difficulty to cope with negative-emotion inducing stimuli, leading to greater stress-induced and emotional eating compared to that in their male counterparts.^19–22^ Overall, preliminary findings suggest that females with obesity are sensitive to food cravings, particularly during menstrual phases, which renders it difficult to successfully diet or lose weight.^23^ Sex differences in obesity have implications for treatment outcomes, as sex differences have also been observed in addictive disorders, including food addiction, and females with obesity generally report poorer treatment adherences and outcomes compared to males with obesity.^23,24^

**Figure 1:**
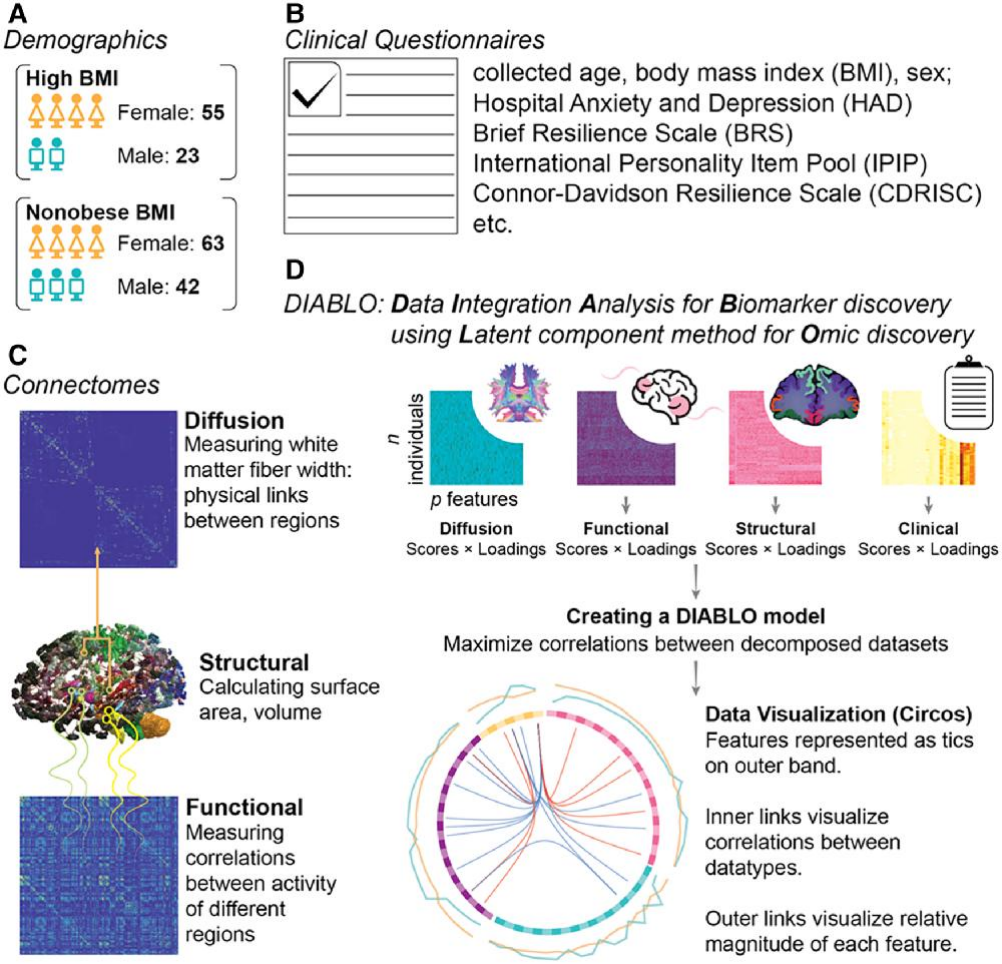
**Study summary figure.** (**A**) Demographics show sample size per group based on sex and BMI status. (**B**) Clinical/behavioural variables were collected from self-report questionnaires. (**C**) Brain structure features from T1 neuroimaging were extracted using FreeSurfer, and connectomes were constructed using resting-state functional and diffusion neuroimaging. (**D**) Data integration analysis for biomarker discovery using latent components was used to create sex-specific multi-modal BMI signatures.

Although previous literature has addressed the association between obesity and brain responses to stimuli, few studies have investigated sex differences in brain signatures in individuals with and without obesity. Given the widespread prevalence and adverse effects of obesity, it is important to understand the differences in the neural correlates of obesity between men and women. A better understanding of differences in brain signatures in obesity can be used to devise better treatment and effective lifestyle interventions. In this study, we aimed to use a data-driven approach, integrating data from multi-modal brain imaging and behavioural data, to determine sex-specific brain-behavioural signatures present in individuals with obesity (as demonstrated in summary Fig. 1).

## Materials and methods

### Participants

On hundred and eighty-three participants, aged 18–55 years, were recruited from community advertisements and enrolled at the G. Oppenheimer Center for Stress and Resilience from 2015 to 2021. All participants classified as overweight/obese had a body mass index (BMI) greater than 25 kg/m^2^ (referred to as high BMI henceforth) and non-obese weight had a BMI between 19 and 20 kg/m^2^ (referred to as non-obese BMI henceforth). Specifics regarding inclusion and exclusion criteria are specified in the Supplementary material. Procedures were in compliance and were approved by the Institutional Review Board at UCLA’s Office of Protection for Research Subjects. Participants provided written informed consent. There were 436 participants potentially eligible, 264 confirmed eligible and 183 included in the study. Participants comprised 42 males with non-obese BMI, 23 males with high BMI, 63 females with non-obese BMI and 55 females with high BMI.

Participants were excluded if they had any major medical/neurological conditions, current or past psychiatric illness, comorbidities such as vascular disease or diabetes, weight loss/abdominal surgeries, pregnancy or breastfeeding, extreme strenuous exercise (>8 h of continuous exercise per week), substance use, tobacco dependence (≥half a pack daily), metal implants, medications that interfere with the central nervous system or regular analgesic use. No participants exceeding 400 pounds were included due to weight constraints of the MRI scanner. Only premenopausal females were included since female sex hormones such as oestrogen are known to effect brain structure and function.

### Study design

In this cross-sectional study, all participants completed multi-modal neuroimaging, including a T1 structural scan, a resting-state blood-oxygen-level-dependent scan and diffusion-weighted imaging scan. High BMI (obese) individuals were defined as those having a BMI greater than or equal to 30 kg/m^2^ and non-obese BMI individuals were those having a BMI between 18.5 and <30 kg/m^2^.^25^

### Behavioural data/clinical questionnaires

Participants were asked to complete a battery of self-report questionnaires: Yale Food Addictions Scale (YFAS),^26^ Pennebaker Inventory of Limbic Languidness (PILL),^27^ Early Trauma Inventory (ETI),^28^ Childhood Traumatic Events Scale (CTES).^29^ Hospital Anxiety/Depression Scale (HADS),^30,31^ State-Trait Anxiety Inventory (STAI),^32^ Visceral Sensitivity Index (VSI),^33^ Perceived Stress Scale (PSS),^34^ Brief Resilience Scale (BRS),^35^ Connor-Davidson Resilience Scale (CD-RISC),^36^ Bowel Symptom Questionnaire (BSQ),^37^ International Personality Item Pool (IPIP),^38^ Patient Reported Outcomes Measurement Information System (PROMIS)^39^ and 12-item Short Form (SF-12) Survey.^40^ See Supplementary material for further details.

### Neuroimaging acquisition and overview of multi-modal neuroimage processing

#### Neuroimaging acquisition

All neuroimaging was conducted at baseline at the Ahmanson-Lovelace Brain Mapping Center on a 3.0 T Siemens Prisma MRI Scanner and a 20-channel head coil. T1 weighted magnetization-prepared radio-frequency pulses and rapid gradient-echo scans acquired to assess brain structure (repetition time (TR): 2300 ms, echotime (TE): 2.98 ms, (longitudinal relaxation time (TI): 900 ms, flip angle: 9˚, field of view: 240 × 256 mm, acquisition matrix: 240 × 256, slice thickness: 1 mm, voxel resolution: 1 × 1 × 1 mm). A 10-minute resting-state functional magnetic resonance imaging (fMRI) scan was acquired to assess resting-state functional connectivity (rs-FC) (TR: 2000 ms, TE: 28 ms, flip angle: 77˚, acquisition matrix: 64 × 64, slice thickness: 4 mm, voxel resolution: 3.44 × 3.44 × 4 mm, 300 volumes). A diffusion-weighted image was acquired to assess white matter anatomical connectivity (64 non-collinear directions, *b* = 1000 s/mm^2^, 9 *b* = 0 s/mm^2^ images, TR: 9500 ms, TE: 88 ms, field of view: 2304 × 2304, acquisition matrix: 128 × 128, slice thickness: 2 mm, spacing between slices: 2 mm). Quality control was conducted based on previously published studies.^41^

#### Structural image processing

Cortical reconstruction and volumetric segmentation was done using the FreeSurfer 6 analysis suite.^42^ All participants’ T1 structural data were first parcellated using the Destrieux cortical atlas^43^ and the Harvard-Oxford sub-cortical^44–47^ atlas. FreeSurfer then computed values of cortical thickness, surface area, mean curvature and volume for cortical regions of interest (ROIs) and volume for sub-cortical ROIs (Supplementary Table 1).

#### Functional image processing

All functional data were run through a preprocessing pipeline for volume-based rs-FC analyses in functional connectivity toolbox (CONN).^48^ All functional data went through realignment and unwarping, slice-timing correction and outlier identification (advanced retrospective technique-based identification of outlier scans for scrubbing. Functional and structural data (T1 scan) were normalized and segmented grey matter, white matter and CSF tissue.^49^ Data were then denoised by using ordinary least squares regression of potential confounding effects and temporal band-pass filtering. The default anatomical component-based noise correction procedure (aCompCor) include noise components from white matter, CSF,^50^ estimated subject-motion parameters,^51^ outlier scans or scrubbing based on frame-wise displacement,^52^ and effect of rest representing potential ramping effects (at the start of the session).^53^ A temporal band-pass filter between 0.008 and 0.09 Hz after regression was used to minimize the influence of physiological, head-motion and other noise sources.^54^ Fisher transformed correlations were computed (*Z*) in CONN between the functional time series of all the parcellated regions done in FreeSurfer to derive a 165 × 165 matrix for each participant. The bottom half of the undirected matrix was then concatenated into a single vector for each subject representing the correlation strength between each ROI pair.

#### Diffusion processing

All diffusion-weighted images were first corrected for eddy current-induced distortions and movement using FSL’s *eddy_correct* tool.^55^ Diffusion images, b-vectors and b-values were then converted to Camino data formats using Camino’s *fsl2scheme* and *image2voxel*.^56^ A diffusion tensor was then fit on the voxel order data in Camino (*wdtfit)* using weighted linear least squares regression.^56^ Deterministic and probabilistic tensor-based approaches have been shown to perform similarly.^57^ Whole-brain deterministic tractography was then performed using the *track* command in Camino Euler algorithm with a step size of 0.5 and curve threshold of 76. Connectivity matrices were constructed using the *conmat* command in Camino, resulting in a 165 × 165 matrix representing the number of streamlines connecting each pair of ROIs computed in the aforementioned FreeSurfer and CONN analyses. Every subject’s matrix went through within-subject normalization by taking a sum of all counts between each ROI, then dividing each pair’s count by that total. The bottom half of the undirected matrix was concatenated into a single vector for each subject representing the connectivity strength between each ROI pair.

Datasets used in the Data Integration Analysis for Biomarker discovery using Latent Components (DIABLO) analysis were created using an ROI approach using the Destrieux cortical and Harvard-Oxford sub-cortical atlases. Measures of brain morphometry, rs-FC and anatomical connectivity were derived for each ROI.^44–47,58^ Each neuroimaging modality was considered as separate datasets. All datasets went through their own specific preprocessing methods prior to the DIABLO analysis.

### DIABLO analysis

A DIABLO analysis was conducted to determine what limited number of correlated neuro-behavioural features would predict participants with high BMI versus non-obese BMI, and males with high BMI versus females with high BMI. Data were split into training and testing datasets at a 80%/20% split, respectively. DIABLO aims to identify a limited number of correlated variables from multiple, matching high-dimensional datasets (Q) to predict an outcome. It extends sparse generalized canonical correlation analysis to a supervised learning framework, which is a generalization of partial least squares (PLS).^59,60^ Prior to the DIABLO analysis, sparse PLS (sPLS) models were run between each dataset (e.g. morphometry versus clinical; resting-state versus diffusion) to understand the overall correlational structure between data types. A data-driven design matrix was created by obtaining correlations from the first component of each individual sPLS model, which guides data integration in DIABLO.^59^ The design matrix is a Q × Q matrix, with values ranging 0–1, representing how correlated each dataset should be considered in the DIABLO algorithm. The final analytical component consists of a model which is a limited number of features across data types that show high correlation with each other. This gives insight not only to which different ‘omic types are relevant, but how features from different ‘omic types interact with each other.

#### Neuroimaging data preparation for DIABLO

In the morphometry datasets, surface area and volume metrics were residualized by the estimated total intracranial volume to control for effects driven by brain size.^61–63^ As the nature of the diffusion dataset can consist of many variables with zeros (i.e. regions that do not share anatomic connections), near zero variance predictors were removed (with the cut-off for the ratio of the most common value to the second most common value being 80/20 and the cut-off for the percentage of distinct values out of the number of samples being 50%), reducing the dataset from 13 530 features to 1613. The final morphometry dataset had 612 features, anatomical connectivity dataset had 1613 features, resting-state connectivity dataset had 13 530 features and clinical dataset had 41 features. All four datasets (including clinical data) were then scaled and centred separately by calculating mean and standard deviation of each vector, then ‘scaling’ each element by subtracting the mean and dividing by the standard deviation. These datasets were entered into subsequent analyses.

#### DIABLO analysis

Following design matrix construction via sPLS analysis across each dataset, a DIABLO model with five components was fit with no variable selection, and global performance was assessed using 10-fold cross-validation and the balanced error rate (BER). The number of components used in the final model was based on the lowest BER and distance metric (maximum distance versus centroids distance versus mahalanobis distance). Manual tuning was then performed on the DIABLO model to determine the number of features for each component needed to obtain the lowest BER and highest balanced accuracy on the unseen testing set. The main output measures for DIABLO include a set of components composed of selected variables from each dataset (i.e. latent variables), and associated loading vectors (i.e. coefficients assigned to each variable in each latent variable). Loading vectors and their absolute values represent the importance of each variable in DIABLO. All loading vectors are obtained so that the covariance is maximized between a linear combination of X (i.e. the different neuroimaging data type variables) variables and Y (i.e. obesity group). Individual sample plots characterize everyone projected onto a space defined by the latent variables. The position on the coordinate plane for everyone is determined by their scores on each component determined by DIABLO. Loading plots show the importance of each variable in each component of each dataset. Circos diagrams represent the correlation between variables from each X data type used in the DIABLO model. A cut-off of *r* = 0.7, representing a ‘strong’ correlation was chosen.^64^ The same cut-off was used to create relevance networks, which represent only the correlated variables in the DIABLO model, allowing for a more digestible interpretation. The area under the receiver operating characteristic (ROC) curve was calculated for each data type, for each component. *P*-values were calculated using the Wilcoxon test comparing non-obese participants versus obese participants and obese male participants versus obese female participants. The area under the ROC curve, which ranges from 0 to 1, where 1 represents a perfectly accurate model, is a way to summarize the overall diagnostic accuracy of the model. A value between 0.7 and 0.8 is considered acceptable, while between 0.8 and 0.9 is considered excellent, and greater values are considered outstanding.^65^

### Data availability

Data can be requested by sending an email to the UCLA G. Oppenheimer Center for Neurobiology of Stress and Resilience (CNSR) at OCNSadmin@mednet.ucla.edu. CNSR may deny requests if they conflict with the Data Sharing plan outlined by the funding source(s) for which the data were obtained. Recipients of approved request must agree to a Data Sharing Agreement, which applies to both the raw data as well as to any new data derived solely or in part from the data received. Any resulting publications utilizing CNSR data should acknowledge the methods of CNSR data gathering using language recommended by CNSR as well as their funding sources.

## Results

### Clinical results

Tables comparing clinical variables between high BMI versus non-obese BMI and males with high BMI versus females with high BMI are depicted in Tables 1 and 2 and Supplementary Tables 2 and 3 (for all sub-scale variables). The distribution of BMI is shown in Supplementary Fig. 1. High BMI individuals had significantly greater scores on the BSQ, the CTES, the ETI, anxiety and depression on the Hospital Anxiety and Depression Scale, the PILL, the VSI and the Yale Food Addiction Scale. High BMI individuals had significantly lower scores on the physical and mental components of the Short Form Health Survey.

**Table 1:** Clinical differences between high BMI and normal BMI

Characteristic	*N*	Non-obese BMI^a^*N* = 105	High BMI^a^*N* = 78	*P*-value^b^	*q*-Value^c^	Cohen’s D (95% CI)^d^
BRS score	180	23.2 (4.2)	22.8 (4.9)	0.6	0.7	0.09 (−0.21, 0.38)
BSQ overall score	19	1.2 (1.6)	4.9 (3.4)	0.006	0.020	−1.2 (−2.3, −0.09)
CD-RISC score	182	78 (12)	79 (13)	0.7	0.8	−0.06 (−0.36, 0.23)
CTES trauma overall	143	3.6 (4.6)	6.3 (6.9)	0.007	0.020	−0.45 (−0.78, −0.12)
ETI total score	180	3.4 (3.6)	5.8 (4.9)	<0.001	0.004	−0.57 (−0.87, −0.27)
HAD anxiety	183	3.5 (3.3)	5.1 (3.5)	0.002	0.013	−0.48 (−0.77, −0.18)
HAD depression	183	1.65 (2.00)	2.45 (2.68)	0.028	0.072	−0.35 (−0.64, −0.05)
IPIP extraversion	181	35 (6)	36 (8)	0.2	0.3	−0.19 (−0.48, 0.11)
IPIP neuroticism	181	21 (6)	22 (7)	0.2	0.3	−0.20 (−0.50, 0.09)
PCS overall	23	10 (12)	7 (10)	0.6	0.7	0.24 (−0.65, 1.1)
Perceived Stress Scale Score	181	11 (6)	12 (6)	0.3	0.4	−0.17 (−0.46, 0.13)
PILL score	176	5.1 (4.5)	8.7 (6.6)	<0.001	0.002	−0.65 (−1.0, −0.34)
PROMIS sleep score	179	45 (8)	45 (9)	0.6	0.7	−0.09 (−0.39, 0.20)
SF12 mental component score	183	53 (6)	50 (9)	0.017	0.045	0.39 (0.09, 0.68)
SF12 physical component score	183	55.0 (3.1)	53.4 (4.3)	0.007	0.020	0.43 (0.13, 0.73)
STAI Trait Anxiety	181	46 (9)	48 (11)	0.2	0.3	−0.19 (−0.48, 0.11)
VSI score	182	5 (9)	8 (10)	0.030	0.073	−0.33 (−0.63, −0.03)
YFAS symptom count	168	1.24 (1.10)	2.07 (1.53)	<0.001	0.002	−0.63 (−0.94, −0.32)

a Mean (SD); *n*/*N* (%). ^b^Welch two-sample *t*-test. ^c^False discovery rate correction for multiple testing. ^d^Cohen’s D (95% CI).

**Table 2:** Clinical differences between high BMI males and high BMI females

Characteristic	*N*	High BMI male^a^*N* = 23	High BMI female^a^*N* = 55	*P*-value^b^	*q*-Value^c^	Cohen’s D (95% CI)^d^
BRS score	77	23.8 (3.5)	22.4 (5.4)	0.2	0.7	0.27 (−0.22, 0.76)
CD-RISC total score	78	81 (12)	78 (14)	0.4	0.7	0.22 (−0.27, 0.71)
CTES trauma overall	76	5.2 (5.3)	6.7 (7.4)	0.3	0.7	−0.22 (−0.73, 0.28)
ETI total score	77	5.7 (5.2)	5.8 (4.9)	>0.9	>0.9	−0.02 (−0.52, 0.47)
HAD anxiety	78	4.2 (2.5)	5.4 (3.8)	0.11	0.7	−0.34 (−0.83, 0.15)
HAD depression	78	1.96 (2.57)	2.65 (2.72)	0.3	0.7	−0.26 (−0.75, 0.23)
IPIP extraversion	77	38 (7)	35 (8)	0.2	0.7	0.34 (−0.15, 0.83)
IPIP neuroticism	77	20 (5)	23 (8)	0.039	0.7	−0.44 (−0.93, 0.05)
Perceived stress score	76	11.4 (6.5)	12.9 (6.3)	0.4	0.7	−0.23 (−0.72, 0.26)
PILL score	77	9 (7)	9 (7)	>0.9	>0.9	−0.03 (−0.52, 0.46)
PROMIS sleep score	78	44 (9)	46 (9)	0.4	0.8	−0.20 (−0.68, 0.29)
SF12 mental component score	78	50 (10)	51 (9)	0.7	0.8	−0.10 (−0.58, 0.39)
SF12 physical component score	78	54.2 (4.6)	53.1 (4.2)	0.3	0.7	0.27 (−0.22, 0.76)
STAI Trait Anxiety	77	48 (9)	48 (12)	>0.9	>0.9	0.01 (−0.48, 0.49)
VSI score	78	8 (8)	9 (11)	0.6	0.8	−0.10 (−0.59, 0.39)
YFAS symptom count	76	1.91 (1.56)	2.13 (1.52)	0.6	0.8	−0.14 (−0.63, 0.35)

a Mean (SD); *n*/*N* (%). ^b^Welch two-sample *t*-test. ^c^False discovery rate correction for multiple testing. ^d^Cohen’s D (95% CI).

### DIABLO results

DIABLO successfully identified a correlated ‘omics signature using multi-modal imaging and clinical and behavioural assessments to classify groups with high BMI from non-obese BMI participants, as well as males with high BMI from females with high BMI. The BER (i.e. the average proportion of wrong classifications in each class validated via 10-fold cross-validation in the training sample) was the critertia for choosing four components for both models. The models achieved AUROC scores of 0.81, *P* = 2.85 × 10^−6^ and 0.91, *P* = 0.002, respectively (Supplementary Tables 4 and 5).

**Figure 2:**
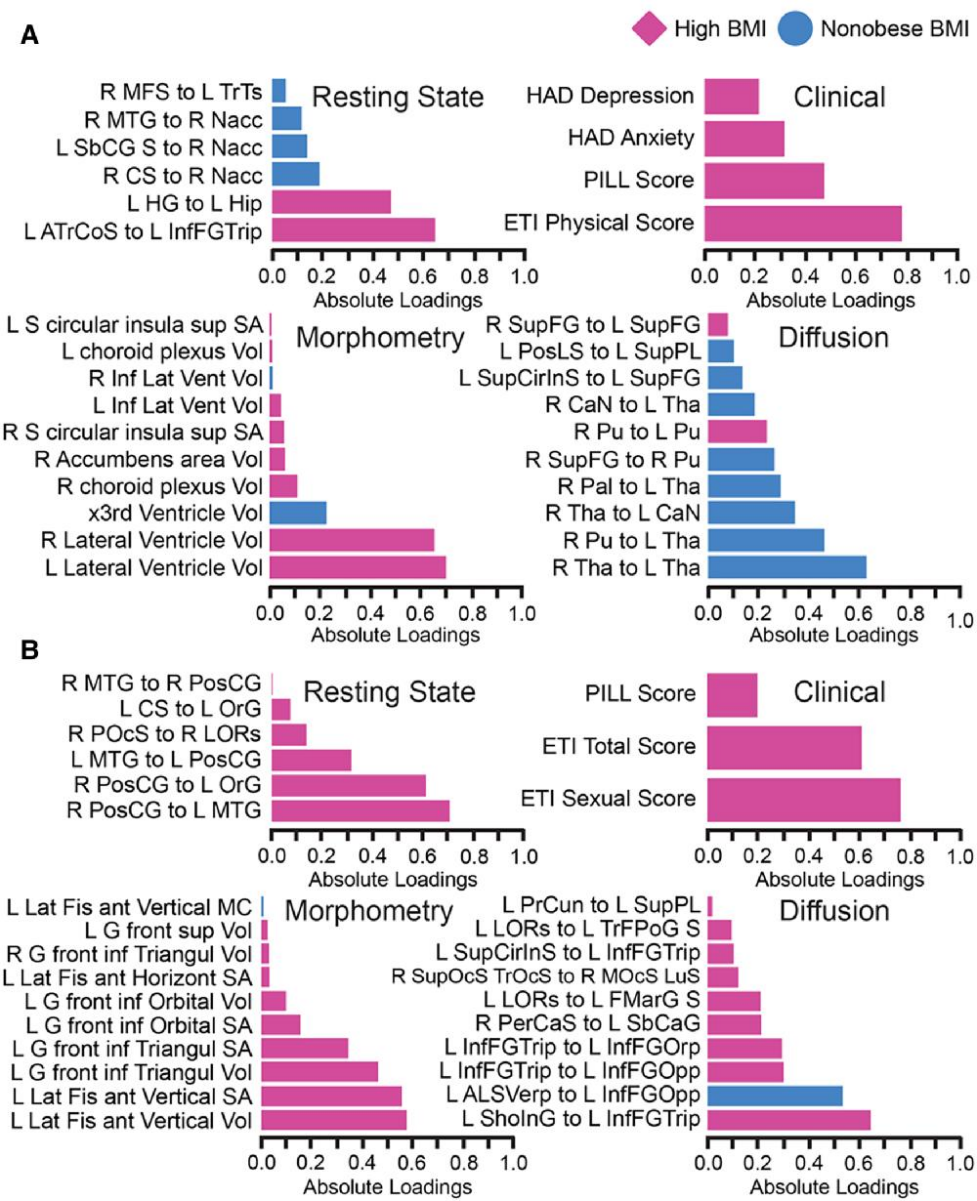
**A highly correlated multi-modal, brain-behavioural signature predicts high BMI (*n* = 78) from non-obese BMI (*n* = 105) individuals via DIABLO.** Absolute loadings depict the relevant importance of each feature. Colours represent which group has a higher mean value of that feature (magenta refers to high BMI and blue refers to non-obese BMI). Sections **A** and **B** represent components 1 and 2, respectively. Region-of-interest labels are provided in Supplementary Table 1.

**Figure 3:**
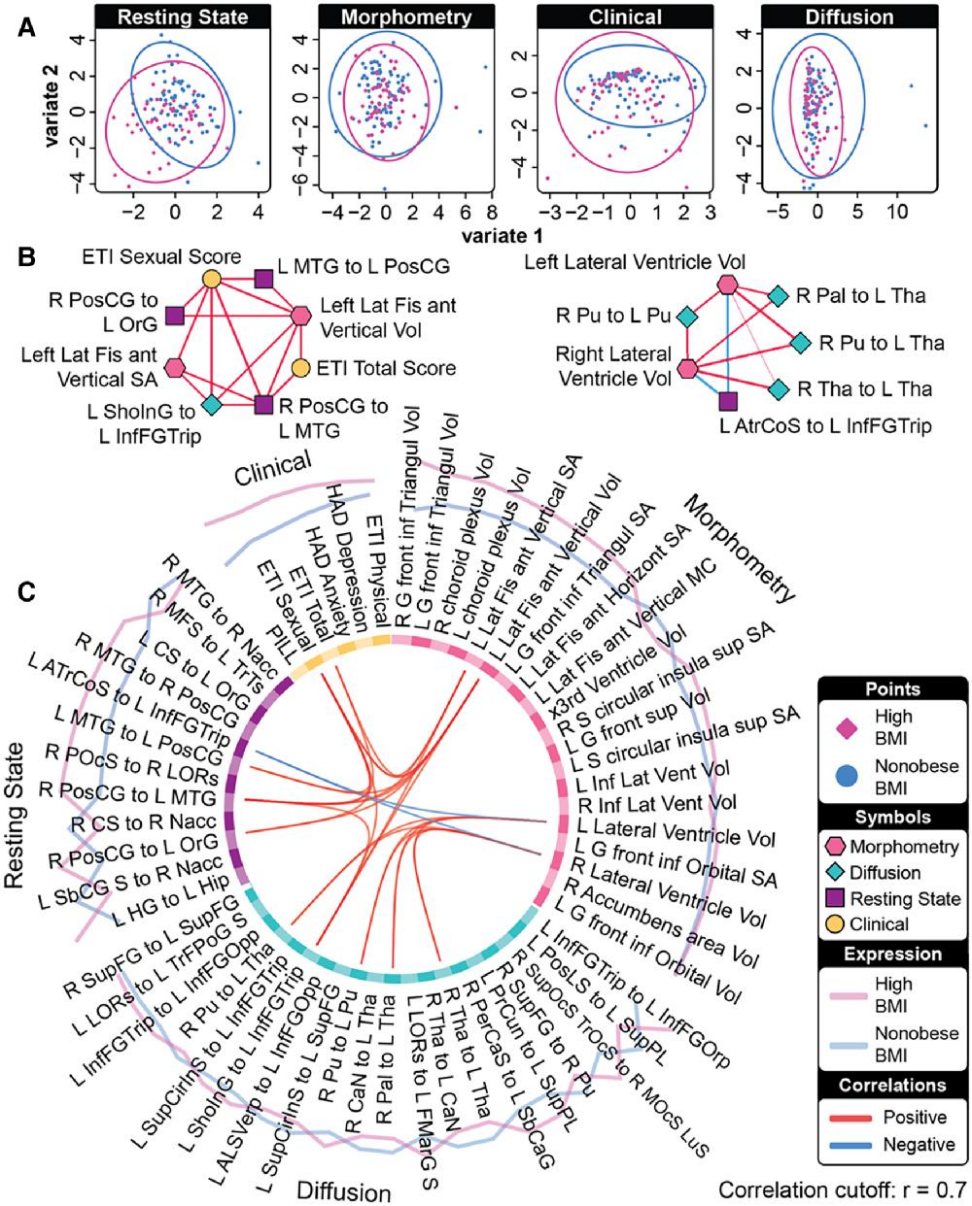
**A combined plot showing characteristics of the model predicting high BMI (*n* = 78) from non-obese BMI (*n* = 105) individuals via DIABLO.** (**A**) Sample plots of clinical, anatomical connectivity, rs-FC and morphometry data. Samples are represented as points according to their projection across the first two latent variables. (**B**) Relevance network represents the absolute Pearson’s correlations between variables of different data types. Magenta lines represent positive and blue lines negative correlations. (**C**) Circos plot representing the correlations between variables of different data types across all four components. Correlation cut-off is set to *r* = 0.7.

For the DIABLO model predicting high BMI versus non-obese BMI participants, the tuning process identified a multi ‘omics signature of four components (Figs 2 and 3A–C).

**Figure 4:**
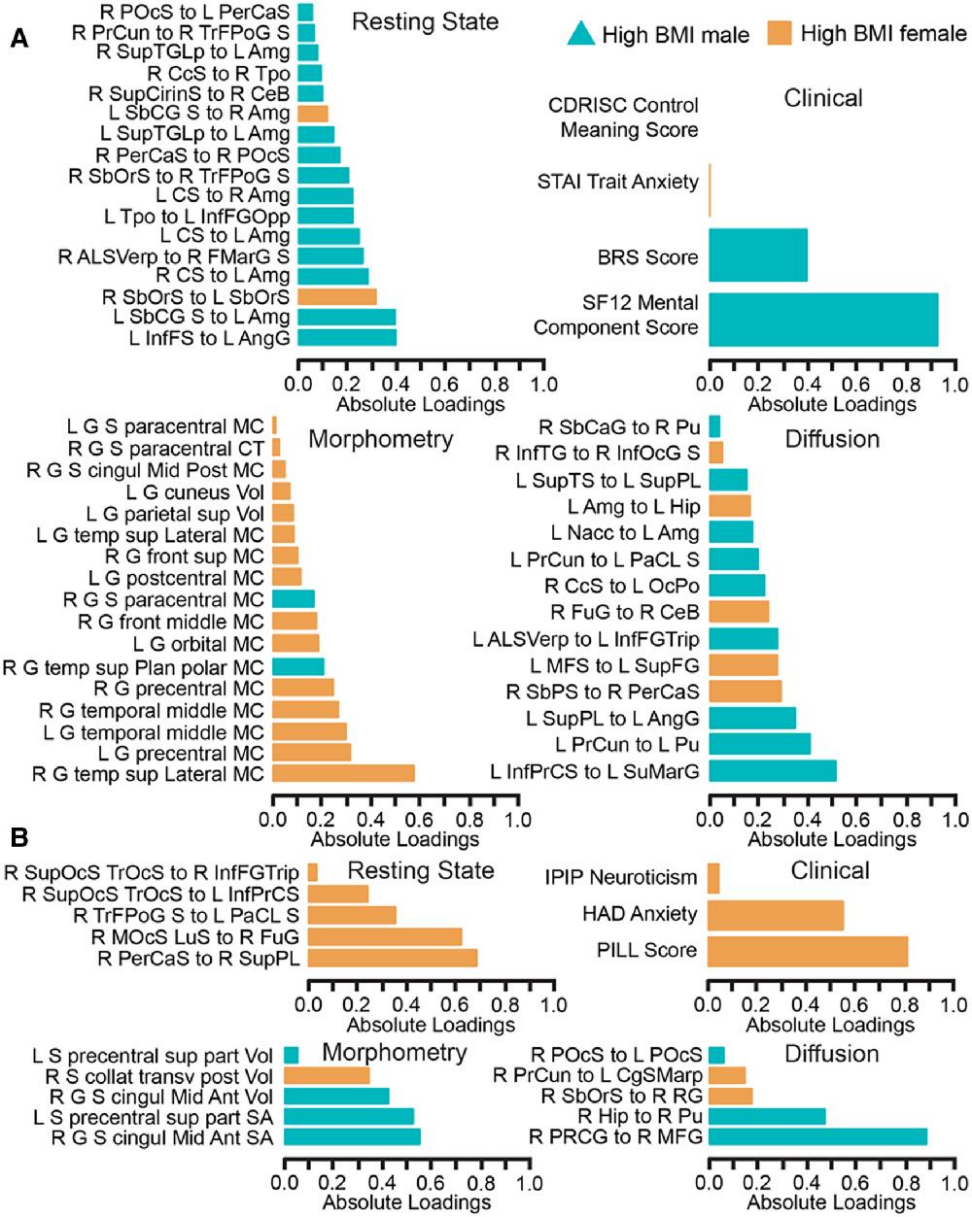
**A highly correlated multi-modal, brain-behavioural signature predicts high BMI males (*n* = 23) from high BMI females (*n* = 55) via DIABLO.** Absolute loadings depict the relevant importance of each feature. Colours represent which group has a higher mean value of that feature. Sections **A** and **B** represent components 1 and 2, respectively. Region-of-interest labels are provided in Supplementary Table 1.

**Figure 5:**
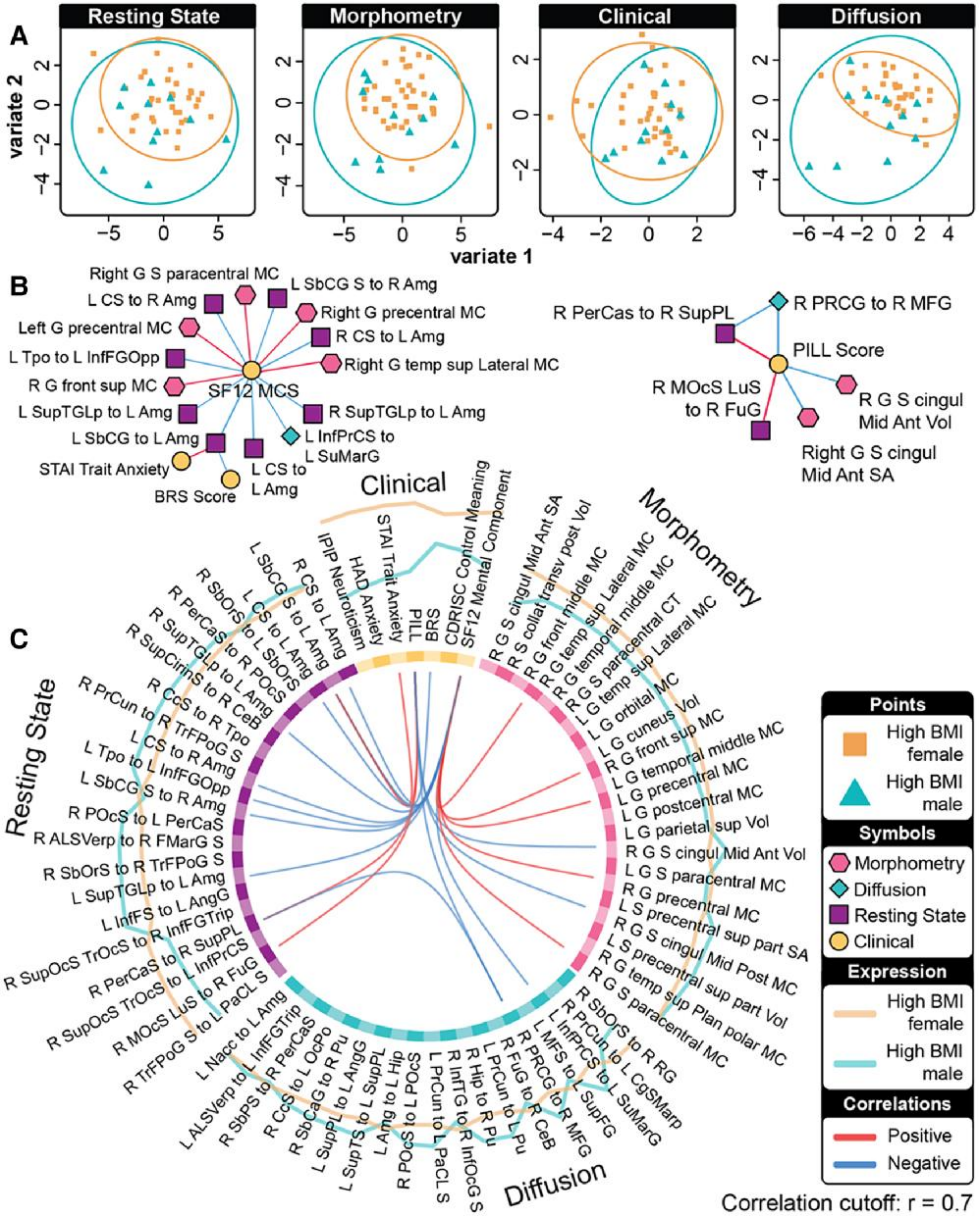
**A combined plot showing characteristics of the model predicting high BMI males (*n* = 23) from high BMI females (*n* = 55) via DIABLO.** (**A**) Sample plots of clinical, anatomical connectivity, rs-FC and morphometry data. Samples are represented as points according to their projection across the first two latent variables. (**B**) Relevance network represents the absolute Pearson’s correlations between variables of different data types. Magenta lines represent positive and blue lines negative correlations. (**C**) Circos plot representing the correlations between variables of different data types across all four components. Correlation cut-off is set to *r* = 0.7.

The signature for the model classifying males with high BMI versus females with high BMI comprised four components (Figs 4 and 5A–C).

**Figure 6:**
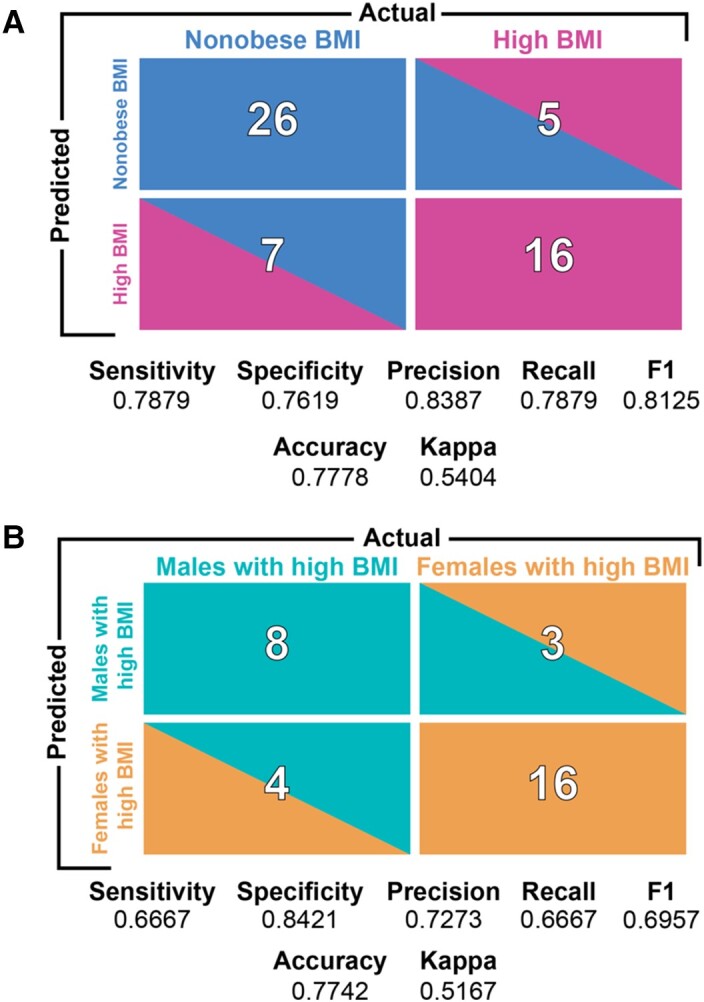
**Confusion matrix statistics on the testing dataset for the model predicting high BMI (*n* = 78) versus Non-obese BMI (*n* = 105), and high BMI males (*n* = 23) versus high BMI females (*n* = 55).** Sensitivity and recall are defined as the true positive rate (i.e. number of predicted improvers divided by the total number of improvers). Specificity is defined as the true negative rate (i.e. number of predicted non-improvers divided by the total number of non-improvers). Precision is the ability of the classifier to not label a true negative as a positive (i.e. the ability to not label a non-improver an improver). The F1 score is the harmonic mean of precision and recall, with values closer to 1 being a better score. Accuracy is defined as the number of true positives and true negatives divided by the total population. The Kappa statistic is known to be a better measure compared to accuracy, especially in the case of imbalanced classes. Kappa values between 0.61 and 0.80 are said to be ‘Substantial’ and between 0.81 and 1.0 to be ‘Almost Perfect’.

Area under the receiver operating curves (AUROC) according to data type and for the final DIABLO model showed high classification accuracy (Supplementary Tables 4 and 5). Predictions and confusion matrix statistics were then assessed using the final model on an external test dataset (Fig. 6).

### Prediction of high BMI and non-obese BMI based on an external test set and contributing features

A DIABLO model trained on training data was used to predict whether groups of participants could be classified as high BMI or non-obese BMI from an independent testing dataset consisting of multi-modal brain and clinical data. The model predicted classes on the test dataset with a balanced accuracy of 77% and *F*1 = 0.81 (Fig. 6A). Features that contributed to the model, the relevance network and the group expressing the maximal value on components 1–2, can be seen in Fig. 3A–C. Sample plots in the two-component space for each data type can be seen in Fig. 3A. The relevance network can be seen in Fig. 3B and circos diagram in Fig. 3C.

### Prediction of males and females with high BMI based on an external test set and contributing features

A DIABLO model was tuned on a training set of participants with high BMI and used to predict whether participants could be classified as male or female on an independent testing dataset consisting of multi-modal brain and clinical data. The model predicted classes with a balanced accuracy of 75% and *F*1 = 0.70 (Fig. 6B). Features that contributed to the model, the relevance network and the group expressing the maximal value on components 1–2, can be seen in Fig. 5A–C. Sample plots in the two-component space for each data type can be seen in Fig. 5A. The relevance network can be seen in Fig. 5B and circos diagram in Fig. 5C.

## Discussion

The objective of the study was to examine sex differences in brain networks related to obesity. The main findings of the study were: (i) greater choroid plexus and ventricular and volume in high BMI individuals was associated with anatomical connectivity alterations in the cortico-basal ganglia-thalamo-cortical loop; (ii) greater early life trauma was associated with greater volume and surface area and anatomical connectivity of the anterior insula and greater sensorimotor–default-mode network resting-state connectivity; (iii) compared to males with high BMI, females with high BMI showed lower mental health scores which was associated with lower amygdala rs-FC to the sensorimotor network and (iv) compared to males with high BMI, females with high BMI had greater PILL scores which were associated with lower surface area and volume in the anterior cingulate cortex. Our results provide a sex-specific biological marker that could explain the short-term feeding regulation and sensory processing patterns seen in females with obesity. To our knowledge, this is the first study to utilize a data-driven approach to predict the sex-specific obesity status of an individual based on multi-modal brain signatures.

### BMI-dependent effects on brain

Individuals with high BMI displayed lower connectivity within the cortico-basal-ganglia-thalamo-cortico (CBGTC) loop, which was associated with greater bilateral lateral ventricle volume. The CBGTC loop plays a key role in the reward circuitry, and in the context of obesity is known to receive dopaminergic input from the ventral tegmental area and substantia nigra to regulate motivational and incentive properties of food.^66^ Altered white matter properties in this loop have been shown repeatedly,^67^ and lower count of white matter tracts within this circuitry can translate to the individual being less efficiently being able to regulate food intake beyond haemostatic needs. Our results showed changes in many regions of the brain which respond to food or food-associated cues^66^ and were highly correlated with early life trauma. One key region was the orbital gyrus, known to play a role in encoding the reward value for food and its greater functional connectivity to the sensorimotor network. The role of the orbital frontal cortex (OFC) in encoding the reward value of palatable food is well established,^66^ but the current results suggest its interactions with the insula can be disrupted by trauma in early life. In the context of childhood maltreatment, the OFC and insula are regions part of an extensive circuitry known to be involved in the threat response.^68^ Repeated exposure is shown to damage this circuitry and promote behaviours that diminish approach responses in concordance with an experience-dependent adaptation hypothesis.^68^ In the context of obesity, those with early life trauma may suggest a risk factor for a consistently active threat response, as observed in individuals with obesity indexed via psychophysiological parameters.^69^

The current results show greater choroid plexus and ventricle volume in those with high BMI. Research of the choroid plexus-CSF system in obesity is still in its infancy, but it is important to note insulin is produced by ependymal cells of the choroid plexus and is regulated by serotonergic signalling.^70^ Moreover, dense expression of leptin receptors in the choroid plexus^71^ and its role in transporting leptin into the brain across the blood brain barrier^72^ suggests the current results of greater choroid plexus and ventricle volume may be directly reflective of the amount of adipose tissue in the body.^73^ Leptin and insulin are known to be very high in obese individuals due to the resistance that develops over time. The body compensates by releasing more of these hormones,^74,75^ which has the potential to be reflected via larger choroid plexus and ventricular volumes, but further research looking at these molecular mechanisms in the brain are needed. Corroborating a recent report of the role of the ventromedial prefrontal cortex (vmPFC)-reward network connectivity being associated with decreased systemic leptin during fasting, our results showed those with high BMI had greater anatomical connectivity between the vmPFC and anterior insula, along with greater surface area of the anterior insula, where much of hedonic value processing is conducted.^66^ Moreover, the current results may suggest accelerated aging in those with high BMI, as greater ventricular volume in the context of aging has been associated with greater BMI.^76^ Obesity is also known to accelerate the aging process at a systemic level,^77^ and the current results provide grounds for investigating the cellular molecular mechanisms underlying brain aging in obesity.^78^

### Sex-specific and BMI effects on brain

Compared to males with high BMI, females with high BMI showed lower scores on the mental health sub-scale of the SF-12, which was highly correlated to lower resting-state connectivity between the amygdala and various regions of the sensorimotor network. These regions of the sensorimotor network also had greater mean curvature. Lower connectivity between the amygdala and sensorimotor network was associated with greater anxiety and lower resilience, both of which were observed in females with high BMI. Regions in the sensorimotor network, have been implicated in inappropriate cognitive evaluations of incoming sensory stimuli, resulting in an increased motivational reward placed on food-related stimuli, especially in females compared to males.^79,80^ The establishment of a amygdala–sensorimotor network through direct anatomical connections measured via diffusion imaging^81^ and a resting functional network through fMRI^82^ suggests that the amygdala ‘works in tandem with cortical sensory/motor areas to facilitate the preparation of adaptive responses to social and affective signals’^81^ and directs goal-directed behaviour. Lower connectivity between the amygdala and this established sensorimotor network in women with high BMI compared to males suggests that females may have a reduced capacity to integrate emotions with action-directed goal planning, resulting in greater ‘emotional overeating’ compared to males.^83,84^ This is further supported as our results show altered anatomical and resting-state connectivity of the amygdala to regions all across the recently discovered extended amygdala–sensorimotor network^82^ including the operculum and temporal cortices, which was all associated with lower mental health scores in females with high BMI compared to males with high BMI.

Females with high BMI compared to males with high BMI also had greater PILL scores associated with lower surface area of the anterior cingulate cortex, a key hub of the salience network. The salience network is responsible for selecting pertinent stimuli, either from the environment or internally, that are context-specific, in order to guide the organism's attention and allocation of resources^85^ The PILL questionnaire is conceptualized as a measure the propensity of an individual to report physical symptoms and a measure of ‘somatic focus’.^86,87^ Females with obesity have been shown to have greater salience network connectivity,^88^ and the neural signatures support the incentive salience model. The incentive salience model states the motivational value for food is based on the sight, smell and taste of ultra-processed foods, and reduces the incentive value for other rewards.^89^ The increased somatic focus and alterations in salience network hubs in females with high BMI further corroborate these past findings.

### Limitations and future directions

Due to the cross-sectional nature of the study, we were unable to address questions of causality between observed brain changes and BMI, which future longitudinal studies will determine whether the brain alterations in patients with obesity reflect a pre-morbid state or whether the brain undergoes structural/functional remodelling due to obesity pathology. Other measures of obesity, apart from BMI, such as visceral adiposity or waist–hip ratio should be used to validate the comprehensive nature of obesity.

### Conclusions and clinical implications

To our knowledge, this is one of the few studies demonstrating an association between sex differences in brain signatures in individuals with high BMI using a multi-modal brain imaging approach.^90,91^ Sex differences reveal that women with obesity have lower connectivity between the amygdala and sensorimotor network associated with early life trauma, and greater somatosensation associated with morphological measures of the salience network. Alterations in these networks suggest that compared to men, women with high BMI have greater vulnerability to develop hypersensitivity and salience to highly palatable foods, and increased alterations in ingestive behaviours such as food cravings and food addiction. Our findings shed light on the importance of personalized treatments for obesity that consider the sex of the affected individual. For example, in females with high BMI it may be more pertinent to consider emotional regulation techniques and vulnerability factors such as early life diversity in treatments focused on food intake.^92,93^ This study contributes to the understanding of the nuances driving the sex-specific pathophysiology of obesity. The ability to predict the sex-specific obesity status of an individual based on neural alterations lends itself to the future of individualized treatment plans and preventative medicine. Although causality is unknown, the strong associations between clinical markers, such as anxiety, depression, obesity and neural signatures suggest the importance of the bidirectional mechanistic connection of the gut–brain axis.

## Supplementary Material

fcad098_Supplementary_Data

## Abbreviations

ART =: advanced retrospective technique
AUROC =: area under the receiver operating characteristic
BMI =: body mass index
BRS =: Brief Resilience Scale
BSQ =: Bowel Symptom Questionnaire
CD-RISC =: Connor-Davidson Resilience Scale
CONN =: CONN functional connectivity toolbox
CTES =: Childhood Traumatic Events Scale
D2 =: dopamine receptors
DIABLO =: data integration analysis for biomarker discovery using latent components
ETI =: early trauma inventory
fMRI =: functional magnetic resonance imaging
HADS =: hospital anxiety/depression scale
IPIP =: international personality item pool
MP-RAGE =: magnetization-prepared radio-frequency pulses and rapid gradient-echo
OFC =: Orbital Frontal Cortex
PCS =: Pain Catastrophizing Scale
PILL =: Pennebaker Inventory of Limbic Languidness
PLS =: partial least squares
PROMIS =: Patient Reported Outcomes Measurement Information System
PSS =: Perceived Stress Scale
ROI =: region of interest
rs-FC =: resting-state functional connectivity
SF-12 =: 12-item Short Form Survey
SMN =: Sensorimotor Network
sPLS =: sparse PLS
STAI =: State-Trait Anxiety Inventory
TE =: echo time
TR =: repetition time
T1 =: (longitudinal relaxation time)
vmPFC =: ventromedial prefrontal cortex
VSI =: Visceral Sensitivity Index
YFAS =: Yale Food Addictions Scale
