# Supplementary material for: Integrated multi-modal brain signatures predict sex-specific obesity status
Source: Brain Commun. 2023 Apr 4;5(2):fcad098. doi: 10.1093/braincomms/fcad098 (PMC10116578; doi:10.1093/braincomms/fcad098)
Supplement: fcad098_Supplementary_Data [file fcad098_supplementary_data.docx]

**Supplementary Methods**

*Behavioral Data/Clinical Questionnaires*

Participants were asked to complete a battery of self-report questionnaires, each of which are described below.

*Ingestive Behaviors*. The Yale Food Addictions Scale (YFAS), a 25-item scale, measures food addiction by assessing signs of substance-dependence symptoms in eating behavior as outlined in the DSM-IV (Gearhardt et al., 2009). The YFAS displayed good internal reliability (α=0.86) in a previous research study based on a 353-respondent survey (Gearhardt et al., 2009). The Pennebaker Inventory of Limbic Languidness (PILL) is a 5-item Likert questionnaire that measures the degree of symptoms and difficulty experienced with swallowing pills (Gearhardt et al., 2012). Past research has demonstrated high internal consistency (Cronbach alpha = 0.85) and strong test-retest reliability (*p* < 0.001) for the PILL (Nativ-Zeltzer et al., 2019).

*Early Life Adversity.* The Early Trauma Inventory (ETI), a 56-item semi-structured interview, measures four domains of childhood traumatic events, including general trauma, physical trauma, emotional trauma, and sexual abuse (Bremner et al., 2000). The ETI has shown to be a valid instrument with high internal consistency (α=0.95) in the assessment of childhood trauma in diverse populations (Bremner et al., 2007). The Childhood Traumatic Events Scale was also used to assess traumatic events that occurred before the age of 17. This includes death of loved ones or close friends, parental divorce or separation, sexual trauma, victim of violence, extreme illness or injury, and other major trauma.

*Mood*. The Hospital Anxiety/Depression Scale (HADS) is a 14-item questionnaire that attempts to identify clinically significant anxiety and depression (Rishi et al., 2017; Zigmond and Snaith, 1983). When applied to samples from the general population, primary care patients, somatic, and psychiatric cases, the HADS has shown strong internal reliability and good concurrent validity (Gupta et al., 2017). The State-Trait Anxiety Inventory (STAI) comprises 40 questions that measure the severity of current symptoms of anxiety and a propensity to be anxious. The STAI has two subscales: one assesses the current state of anxiety, (i.e. how the participant subjectively feels in the moment); the other assesses stable aspects (i.e. relative proneness to anxiety) (Julian, 2011). The STAI has shown high internal consistency (α=0.87–0.93) in several validation studies (Fountoulakis et al., 2006; Gustafson et al., 2020; Iwata et al., 1998; Lee and Mok, 2011; Quek et al., 2004). The Visceral Sensitivity Index (VSI) comprises 15 items and measures different aspects of fear and anxiety that accompany gastrointestinal discomfort and gastrointestinal-specific anxiety (Pletikosic Toncic and Tkalcic, 2017). The VSI has demonstrated high internal consistency (*α* =0.93) and high test-retest reliability, with an inter-class coefficient of 0.86 (Labus et al., 2004). The Perceived Stress Scale (PSS), a 14-item questionnaire, measures the perception of stress by asking about feelings and thoughts based on stressful incidents in order to evaluate the degree to which situations in one’s life are considered stressful (Baik et al., 2019). High scores on the PSS hare correlated with higher levels of biomarkers of stress, such as increased cortisol levels (Walvekar et al., 2015).

*Resilience* The Brief Resilience Scale (BRS) is a concise questionnaire comprising 6 items that measure the ability to recover from stress despite significant experiences of adversity (Fung, 2020). The BRS has been used in research studies in cancer patients, vocational rehabilitation service recipients, and the general adult population, and has demonstrated moderate internal consistency (α = 0.71) (Chmitorz et al., 2018; Fung, 2020; Kunzler et al., 2018; Tansey et al., 2015). The Connor-Davidson Resilience Scale (CD-RISC) is another renowned, but longer, scale (25 items, with each rated on a 5-point scale) that is used to quantify resilience, and is viewed as measure of stress coping ability. The CD-RISC has demonstrated sound psychometric validity, with higher scores reflecting greater resilience and ability to cope with stress (Connor and Davidson, 2003). The International Personality Item Pool (IPIP) was used to measures personality factors of neuroticism and extraversion (Goldberg et al., 2006).

*Physical Health* The Bowel Symptom Questionnaire (BSQ) measures the overall symptom severity in IBS and abdominal pain over the past 1 week (Talley et al., 1995). Uses a 0-20 rating scale (ranging from none to most intense imaginable), and a 5-point scale for the usual severity of IBS symptoms (How bad are your symptoms usually? 1=none, 2=mild, 3=moderate, 4=severe, 5=very severe) (Park et al., 2018). The Patient Reported Outcomes Measurement Information System (PROMIS) is a set of questionnaires developed by the National Institute of Health initiative (NIH) to measure symptoms related to the daily functioning of patients within physical, mental, and social health domains, and can be applied to design treatment plans for various chronic conditions (Gibbons et al., 2020). Finally, the 12-item Short Form (SF-12) Survey assesses physical, emotion, and mental health across eight major health domains (Larson, 2002). It is a widely used research tool that is more practical to use in populations with a limited attention span or mental health conditions compared to the SF-36 (Huo et al., 2018).

*sPLS between datasets for Nonobese and Obese Analysis & Obese Male and Obese Female Analysis*

In order to understand what features would be useful in classifying obese participants from nonobese participants and obese male participants from obese female participants, as well as to guide data integration, a sPLS analysis was completed with each dataset in a pairwise manner (i.e. morphometry vs. anatomical connectivity, morphometry vs resting-state-functional connectivity, anatomical connectivity, vs. resting-state functional connectivity, clinical vs. morphometry, clinical vs. anatomical connectivity, clinical vs. resting-state functional connectivity). A canonical mode was used in sPLS to model a bi-directional relationship between the two datasets. The output from the sPLS includes a set of components (i.e. latent variables), a set of loading vectors, and a list of selected variables. Arrow plots and loadings plots are shown to ease the interpretation between the two types of variables from each dataset. Arrow plots project the samples onto a space which is an overlap of the X and Y (i.e. both datasets) representation plots. Each arrow joins the individual sample from X space to Y space. A short arrow represents a good agreement with both datasets via sPLS. Loading plots help visualize each coefficient (i.e. importance) assigned to the variables in each component of each dataset. Loadings are the coefficients assigned to each variable to define each component, and their absolute values represent the importance of each variable in the sPLS. It is important to note that each loading vector is assigned to a particular component, and the loading vectors are obtained so that the covariance between a linear combination of X variables and Y variables is maximized.

The design matrices for data integration using DIABLO were determined via this sPLS approach by modelling pairwise associations. The correlation between the first components between both datasets in each model were computed (**Figures S7 and S8**).

**Supplementary Results**

*Distribution of BMI across groups*

The distribution of BMI across groups of interest are shown in **Supplementary Figure 1**.

**Supplementary Figure 1**


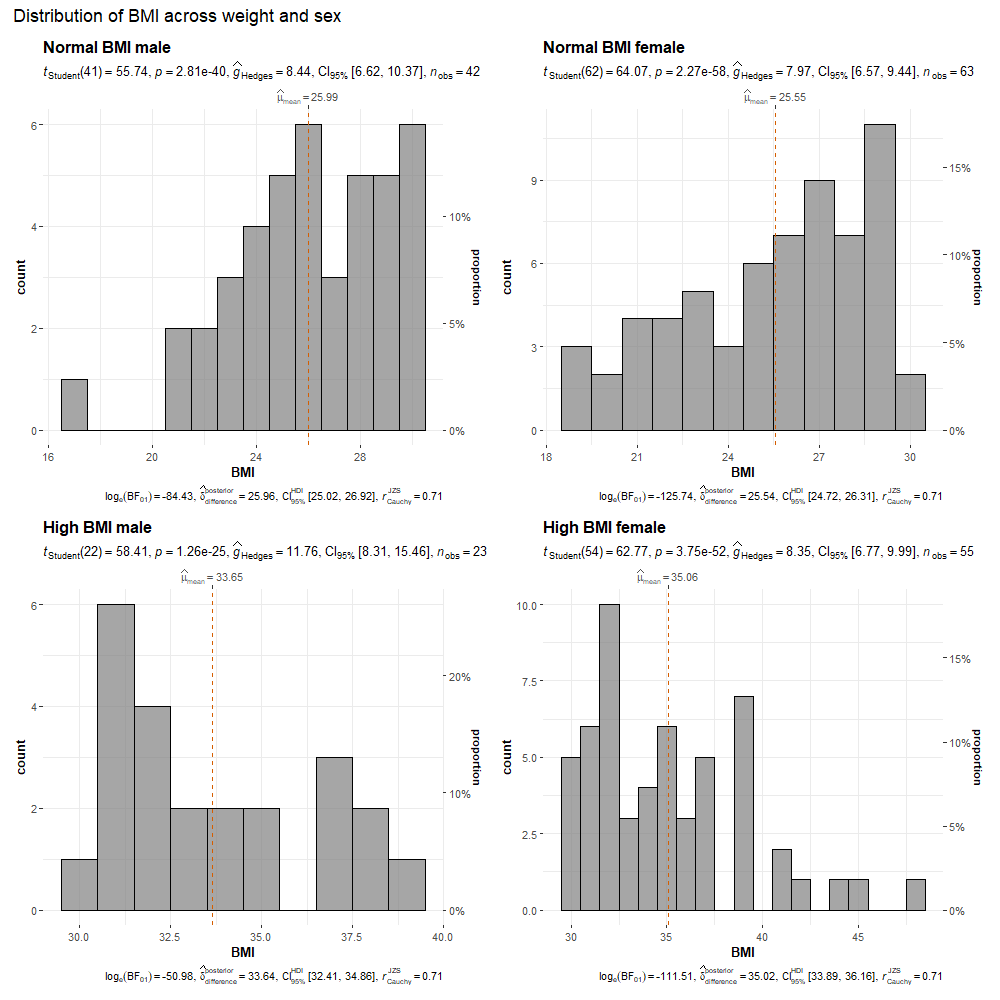


Supplementary Figure 1: Distribution of BMI across each of the 4 groups in the current analyses.

*sPLS between morphometry and anatomical connectivity data for nonobese and obese participants*

A two component sPLS model was used with 50 features from each dataset on each component. The correlation between the first components of the two datasets was r*_(127)_* = 0.839,
p < 0.05, and thus the weight of the design matrix in the DIABO model classifying nonobese participants and obese participants was included. Plots to guide data integration are shown below in **Supplementary Figure 2**.

**
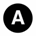

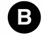

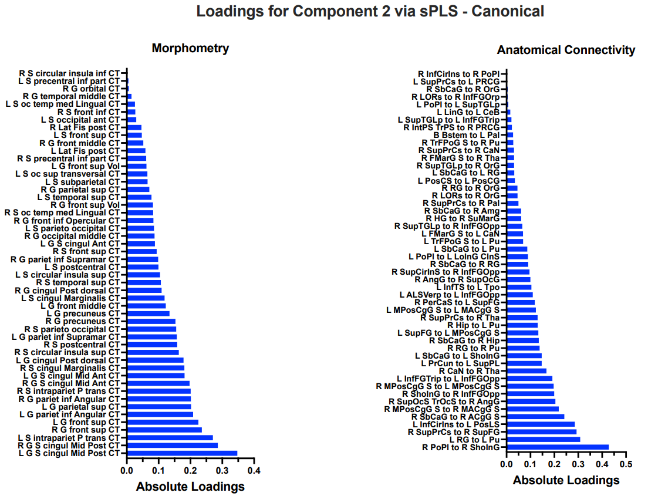

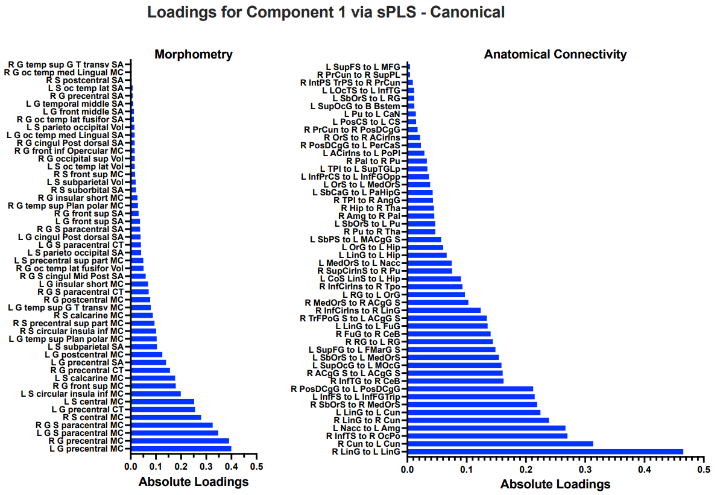
 Supplementary Figure 2**

Supplementary Figure 2: (A) Loadings plots for component 1 per data type, (B) Loadings plots for component 2 per data type.

*sPLS between morphometry and resting-state functional connectivity data for nonobese and obese participants*

A two component sPLS model was used with 50 features from each dataset on each component. The correlation between the first components of the two datasets was r*_(127)_* = 0.688,
p < 0.05, and thus the weight of the design matrix in the DIABLO model classifying nonobese participants and obese participants was included. Plots to guide data integration are shown below in **Supplementary Figure 3**.

**Supplementary Figure 3**


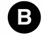

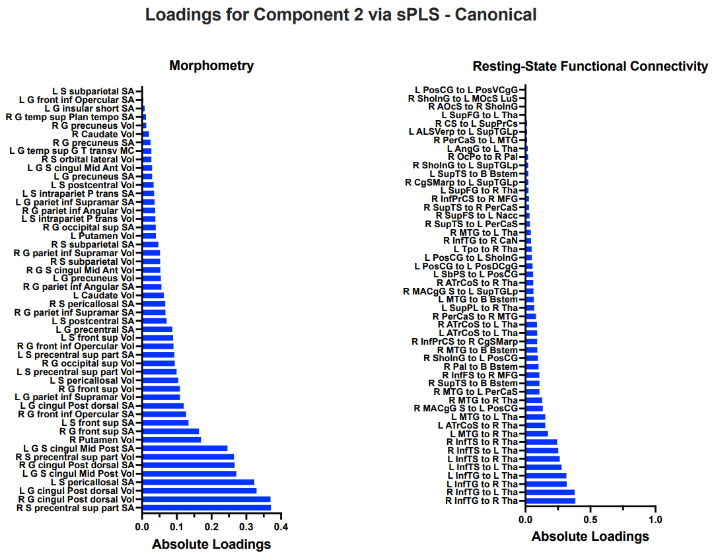

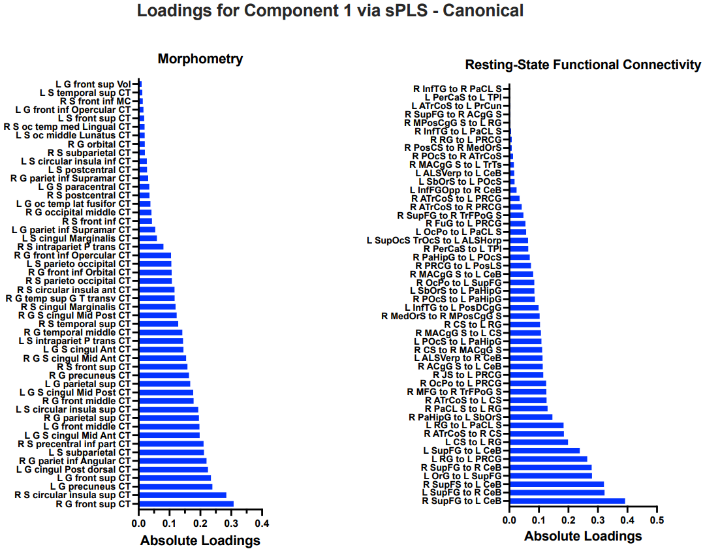

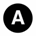


Supplementary Figure 3: (A) Loadings plots for component 1 per data type, (B) Loadings plots for component 2 per data type.

*sPLS between anatomical connectivity and resting-state functional connectivity data for nonobese and obese participants*

A two component sPLS model was used with 50 features from each dataset on each component. The correlation between the first components of the two datasets was r*_(127)_* = 0.0219, p > 0.80. The weight of the design matrix in the DIABO model classifying nonobese participants and obese participants was included. Plots to guide data integration are shown below in **Supplementary Figure 4**.

**Supplementary Figure 4**


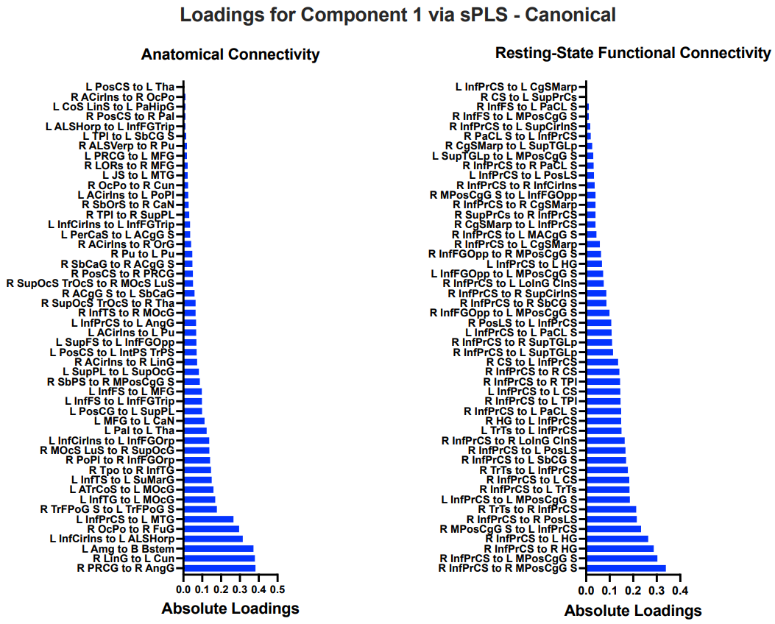

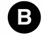

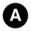

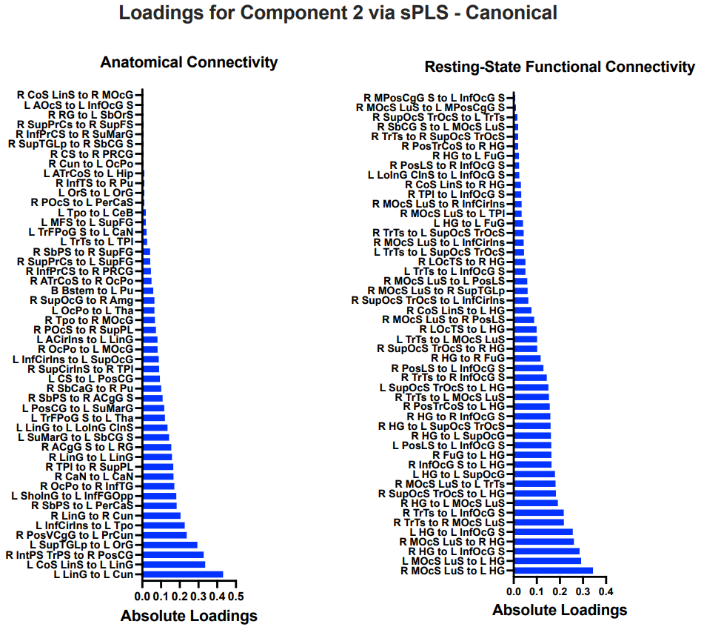


Supplementary Figure 4: (A) Loadings plots for component 1 per data type, (B) Loadings plots for component 2 per data type.

*sPLS between morphometry and clinical data for nonobese and obese participants*

A two component sPLS model was used with 30 features from each dataset on each component. The correlation between the first components of the two datasets was r*_(127)_* = 0.566, p < 0.05, and thus the weight of the design matrix in the DIABO model classifying nonobese participants and obese participants was included. Plots to guide data integration are shown below in **Supplementary Figure 5**.

**Supplementary Figure 5**

*
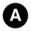

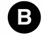
*
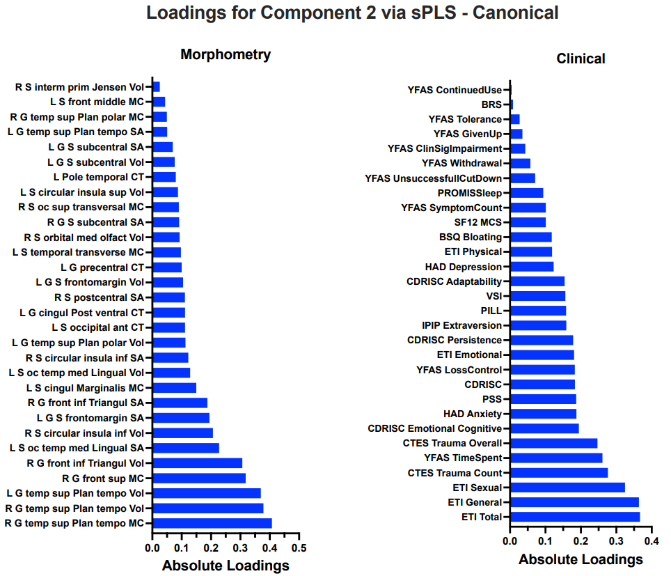

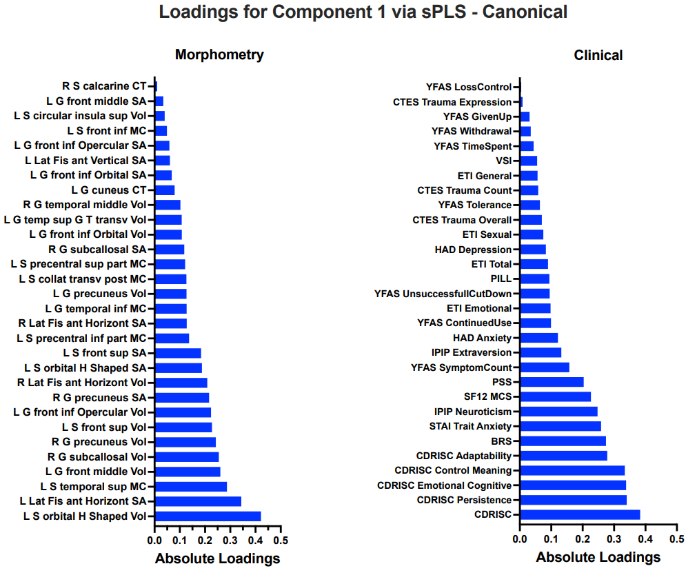


Supplementary Figure 5: (A) Loadings plots for component 1 per data type, (B) Loadings plots for component 2 per data type.

*sPLS between anatomical connectivity and clinical data for nonobese and obese participants*

A two component sPLS model was used with 30 features from each dataset on each component. The correlation between the first components of the two datasets was r*_(127)_* = 0.758, p < 0.05, and thus the weight of the design matrix in the DIABO model classifying nonobese participants and obese participants was included. Plots to guide data integration are shown below in **Supplementary Figure 6**.

**
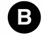

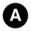

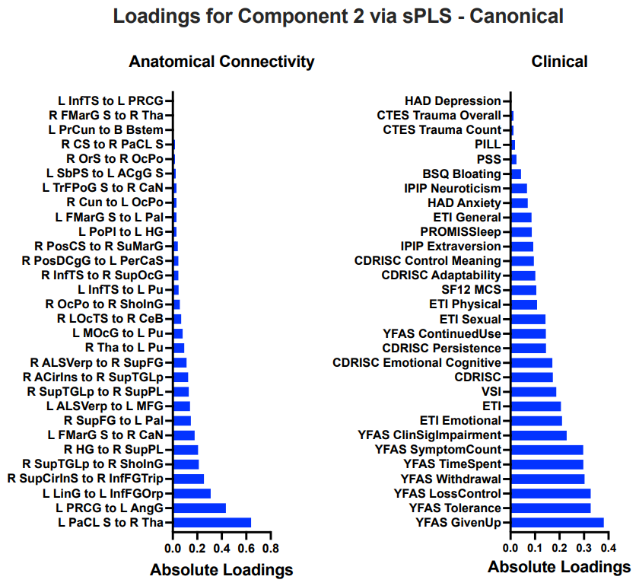

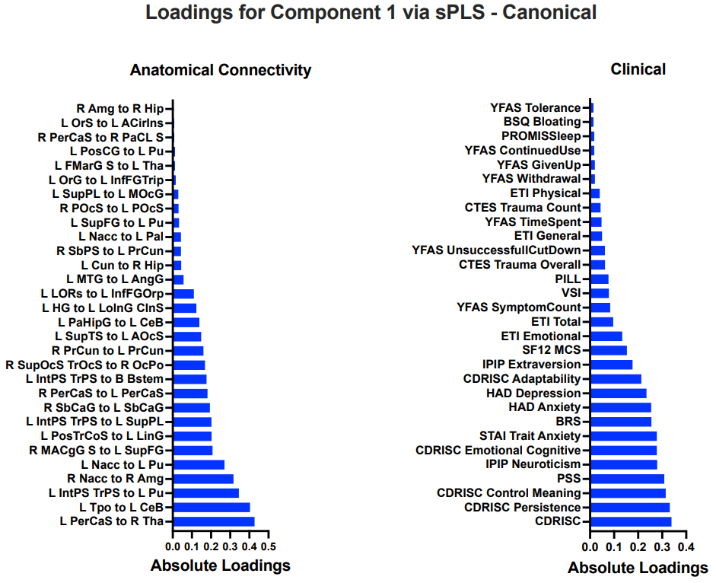
Supplementary Figure 6**

Supplementary Figure 6: (A) Loadings plots for component 1 per data type, (B) Loadings plots for component 2 per data type.

*sPLS between resting-state functional connectivity and clinical data for nonobese and obese participants*

A two component sPLS model was used with 30 features from each dataset on each component. The correlation between the first components of the two datasets was r*_(127)_* = 0.642, p < 0.05, and thus the weight of the design matrix in the DIABO model classifying nonobese participants and obese participants was included. Plots to guide data integration are shown below in **Supplementary Figure 7**.

**Supplementary Figure 7**


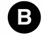

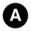

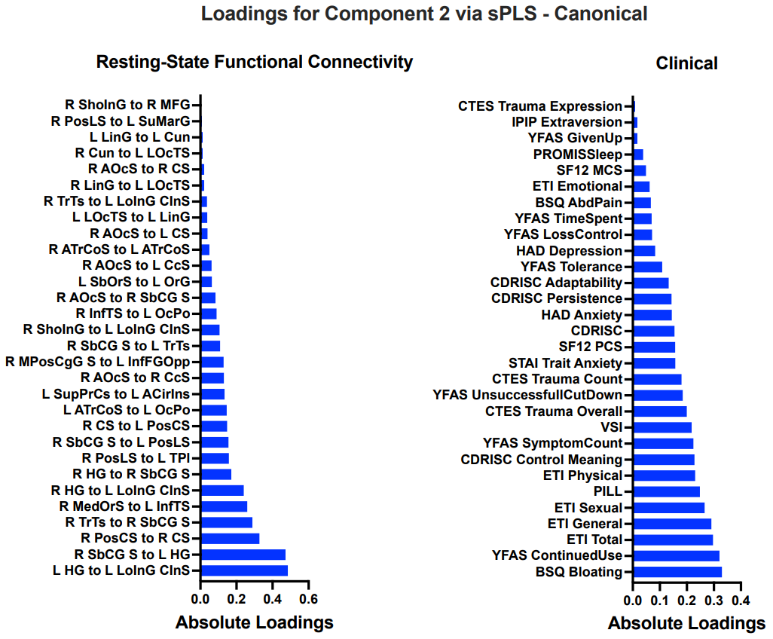

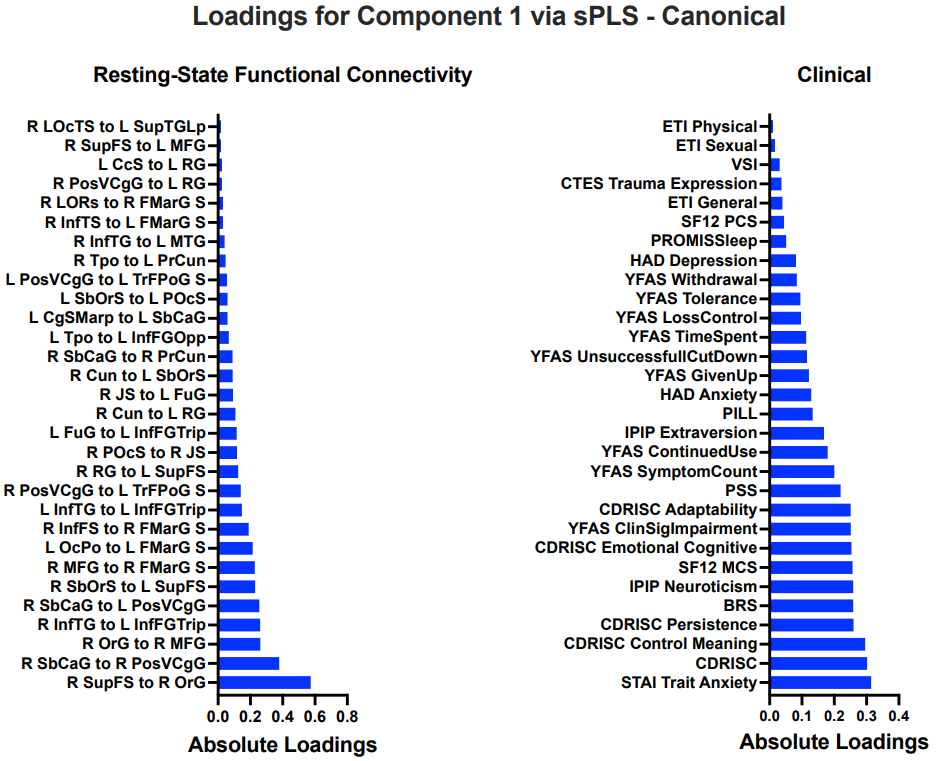


Supplementary Figure 7: (A) Loadings plots for component 1 per data type, (B) Loadings plots for component 2 per data type.

*sPLS between morphometry and anatomical connectivity data for obese male and obese female participants*

A two component sPLS model was used with 50 features from each dataset on each component. The correlation between the first components of the two datasets was r*_(45)_* = 0.915, p < 0.05, and thus the weight of the design matrix in the DIABO model classifying nonobese participants and obese participants was included. Plots to guide data integration are shown below in **Supplementary Figure 8**.

**Supplementary Figure**
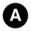

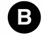

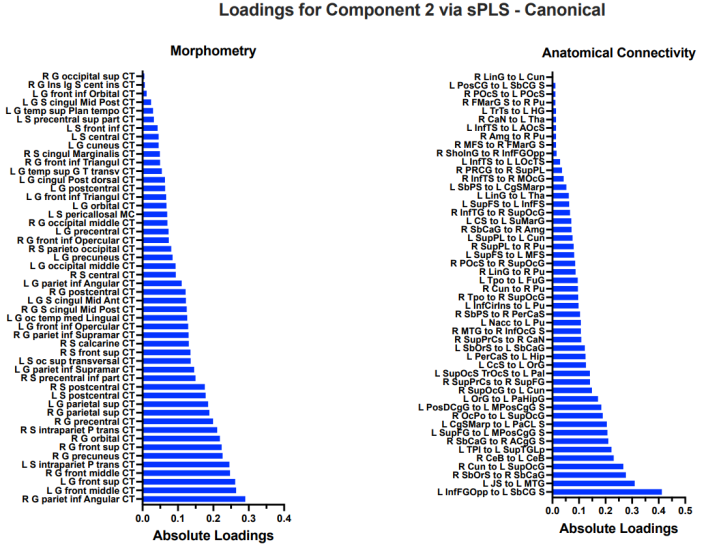

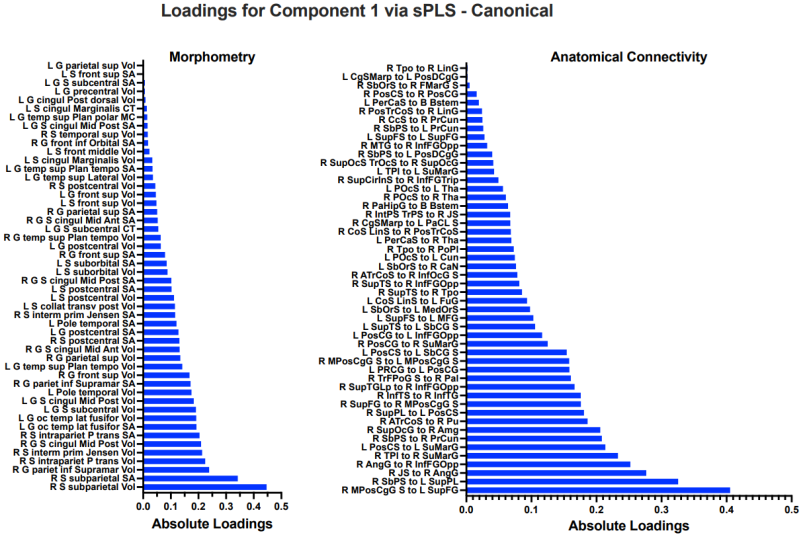
**8**

Supplementary Figure 8: (A) Loadings plots for component 1 per data type, (B) Loadings plots for component 2 per data type.

*sPLS between morphometry and resting-state functional connectivity data for obese male and obese female participants*

A two component sPLS model was used with 50 features from each dataset on each component. The correlation between the first components of the two datasets was r*_(45)_* = 0.832, p < 0.05, and thus the weight of the design matrix in the DIABO model classifying nonobese participants and obese participants was included. Plots to guide data integration are shown below in **Supplementary Figure 9**.

**Supplementary Figure *
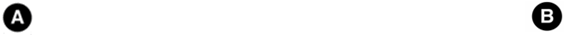

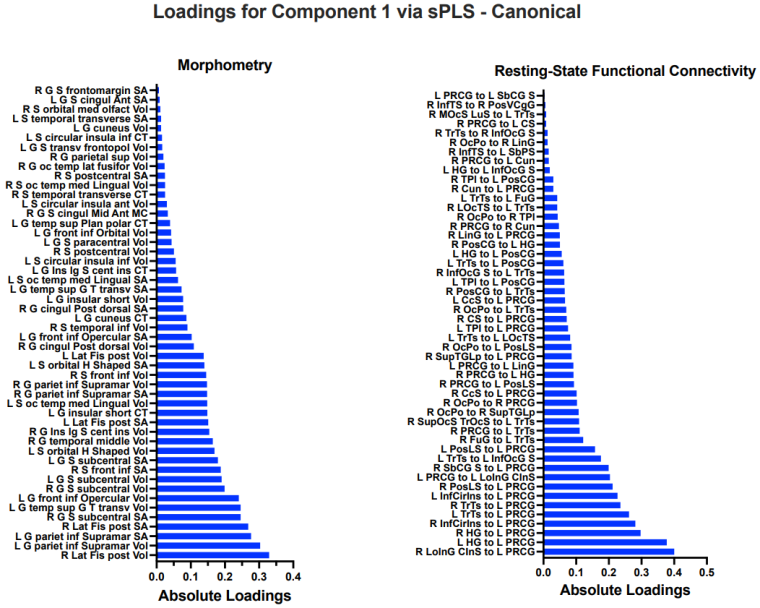

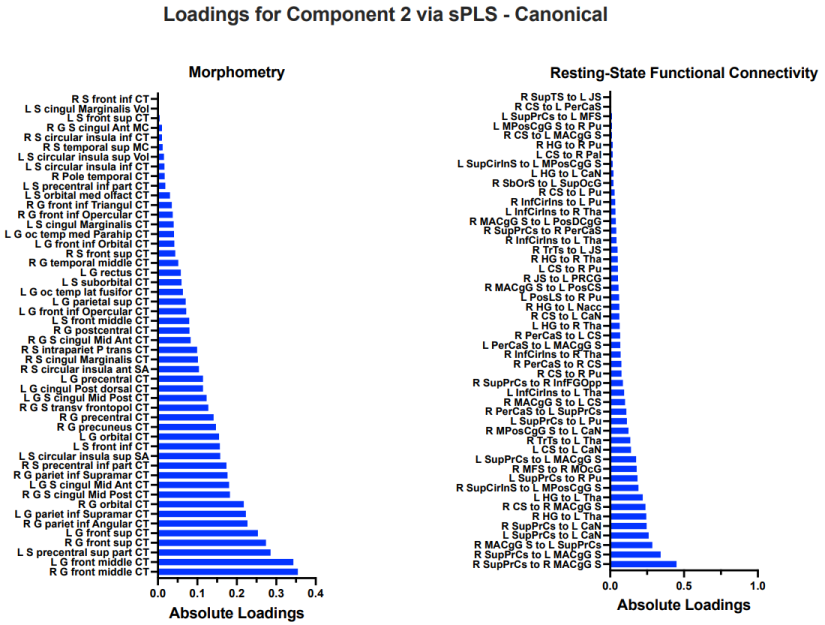
*9**

Supplementary Figure 9: (A) Loadings plots for component 1 per data type, (B) Loadings plots for component 2 per data type.

*sPLS between anatomical connectivity and resting-state functional connectivity data for obese male and obese female participants*

A two component sPLS model was used with 50 features from each dataset on each component. The correlation between the first components of the two datasets was r*_(45)_* = 0.0, p > 0.95. The weight of the design matrix in the DIABO model classifying nonobese participants and obese participants was included. Plots to guide data integration are shown below in **Supplementary Figure 10**.

**Supplementary Figure *
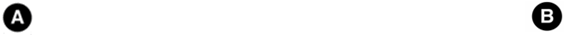

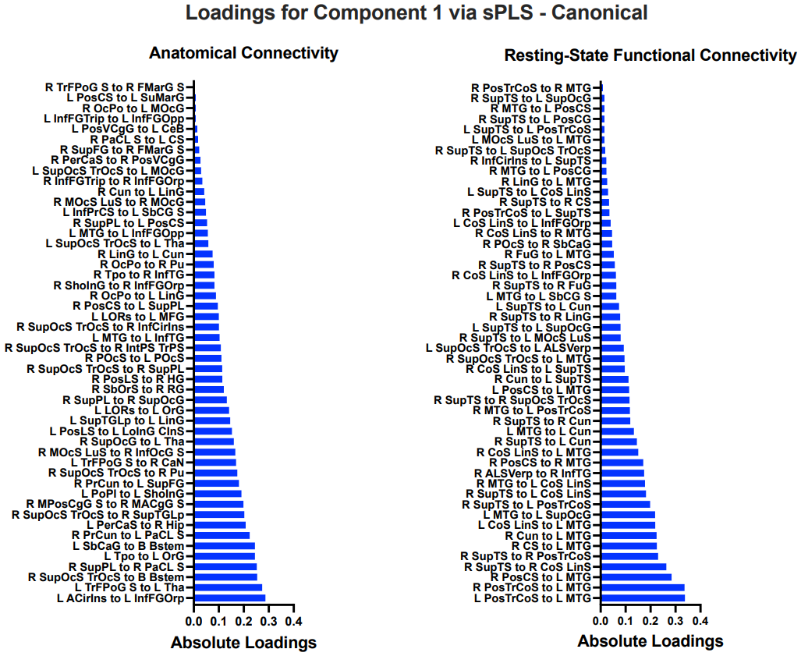

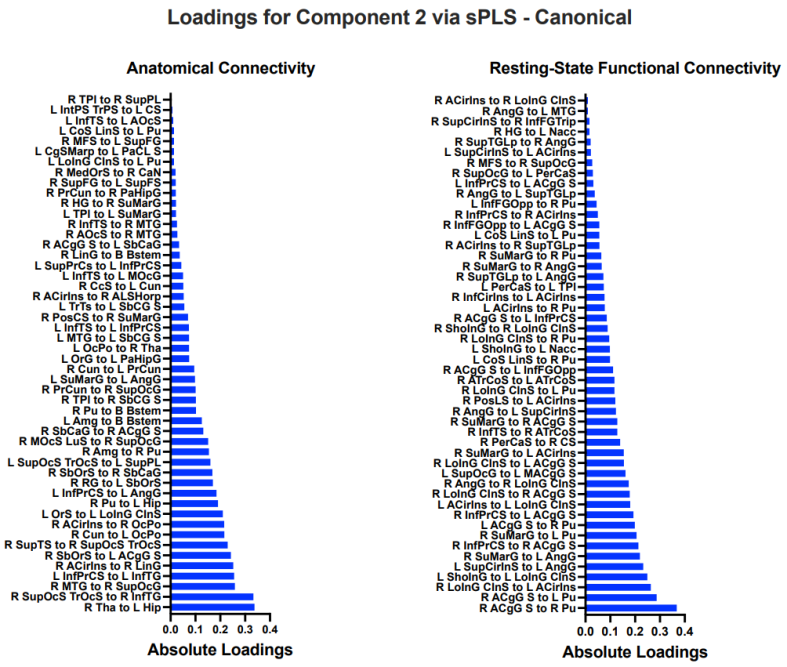
*10**

Supplementary Figure 10: (A) Loadings plots for component 1 per data type, (B) Loadings plots for component 2 per data type.

*sPLS between morphometry and clinical data for obese male and obese female participants*

A two component sPLS model was used with 50 features from the morphometry dataset and 20 features from the clinical dataset on each component. The correlation between the first components of the two datasets was r*_(45)_* = 0.795, p < 0.05, and thus the weight of the design matrix in the DIABO model classifying nonobese participants and obese participants was included. Plots to guide data integration are shown below in **Supplementary Figure 11**.

**Supplementary Figure 11**


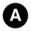

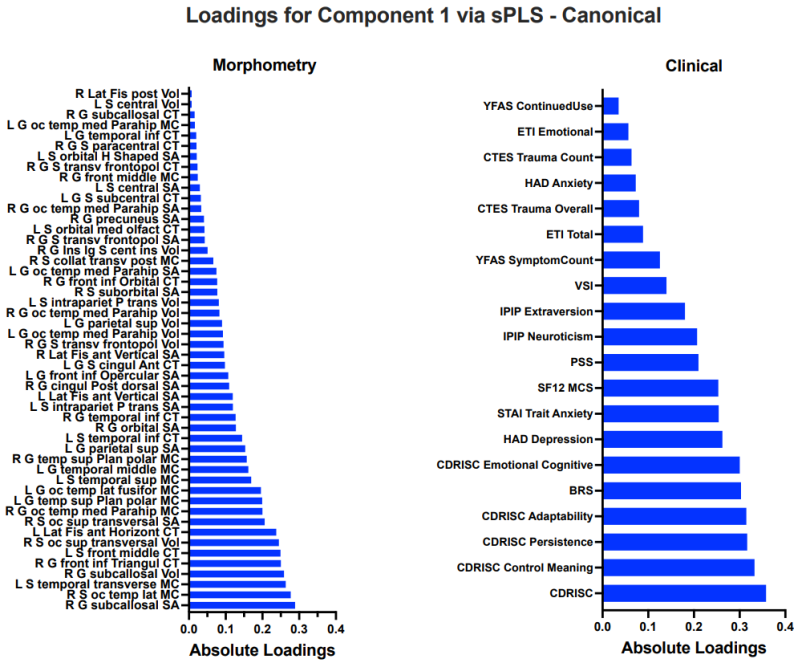

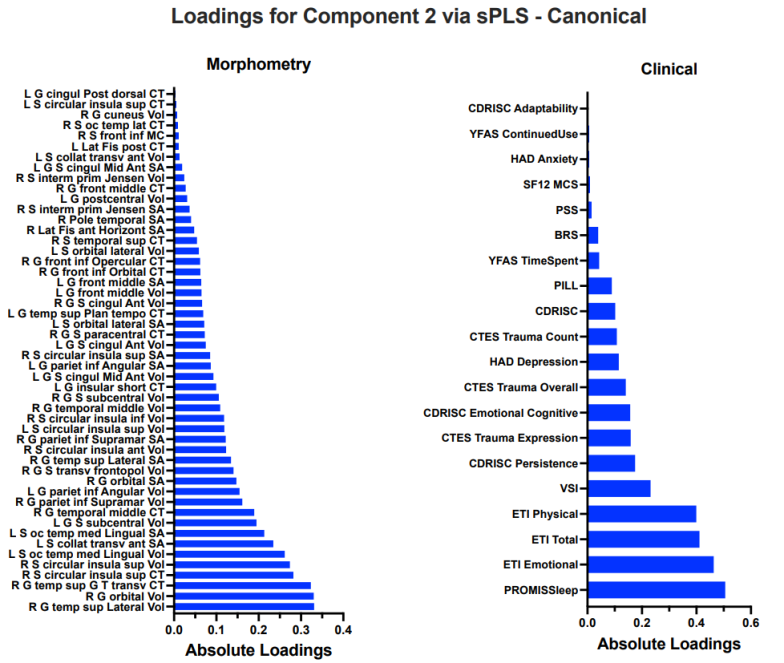

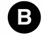


Supplementary Figure 11: (A) Loadings plots for component 1 per data type, (B) Loadings plots for component 2 per data type.

*sPLS between anatomical connectivity and clinical data for obese male and obese female participants*

A two component sPLS model was used with 50 features from the anatomical connectivity dataset and 20 features from the clinical dataset on each component. The correlation between the first components of the two datasets was r*_(45)_* = 0.916, p < 0.05, and thus the weight of the design matrix in the DIABO model classifying nonobese participants and obese participants was included. Plots to guide data integration are shown below in **Supplementary Figure 12**.

**Supplementary Figure 12**


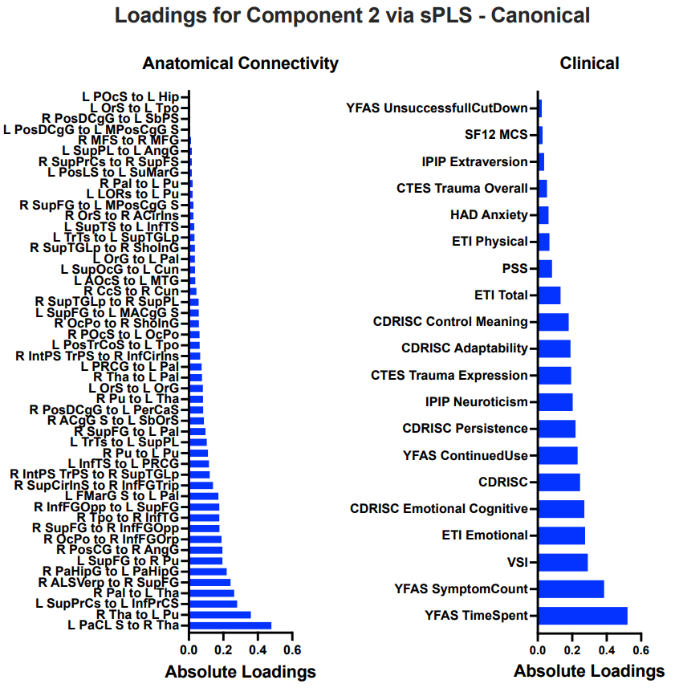

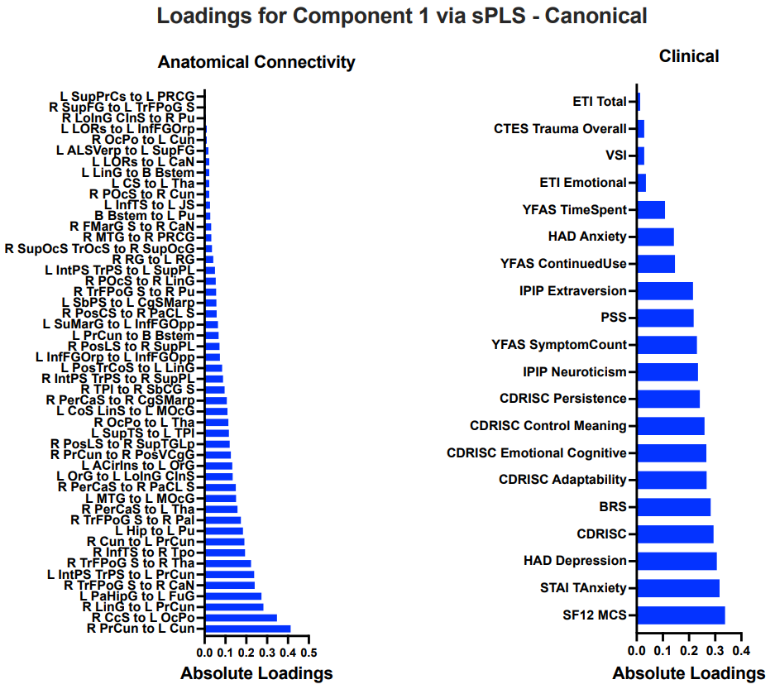

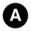

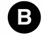


Supplementary Figure 12: (A) Loadings plots for component 1 per data type, (B) Loadings plots for component 2 per data type.

*sPLS between resting-state functional connectivity and clinical data connectivity data for obese male and obese female participants*

A two component sPLS model was used with 50 features from the resting-state functional connectivity dataset and 20 features from the clinical dataset on each component. The correlation between the first components of the two datasets was r*_(127)_* = 0.795, p < 0.05, and thus the weight of the design matrix in the DIABO model classifying nonobese participants and obese participants was included. Plots to guide data integration are shown below in **Supplementary Figure 13**.

**Supplementary Figure *
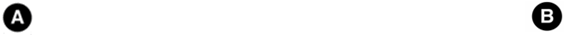
***
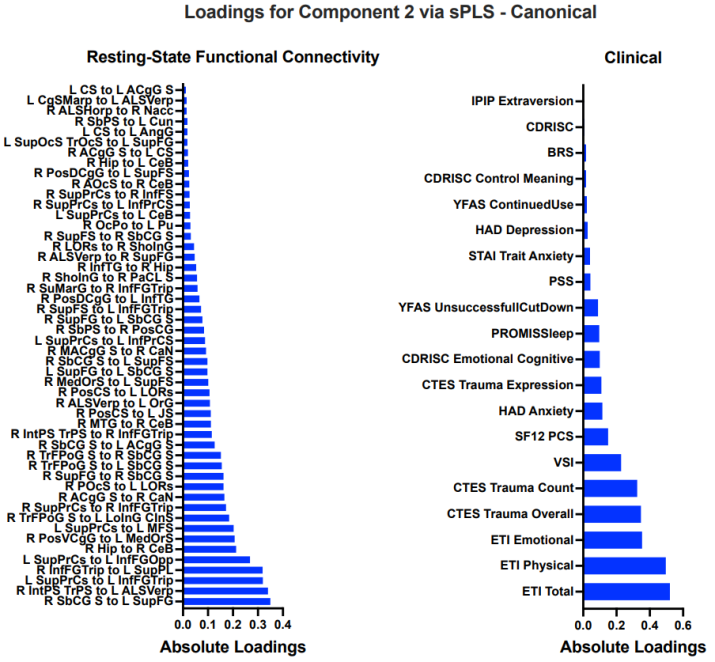
***
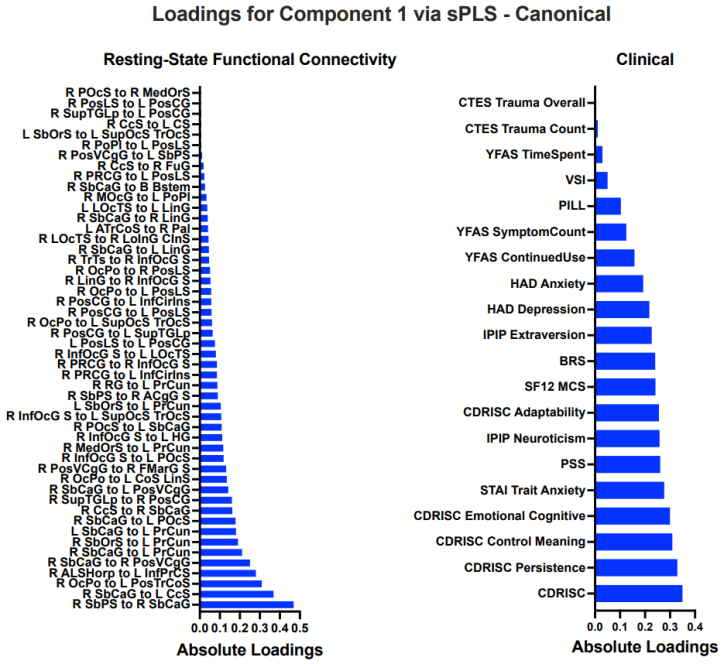
*13**

Supplementary Figure 13: (A) Loadings plots for component 1 per data type, (B) Loadings plots for component 2 per data type.

*Constructing the data-driven design matrices*

In order to determine the design matrix for the DIABLO analysis, a data-driven approach was used. sPLS models were run on each data pair and the correlation between the first component of each dataset was used.

**Supplementary Figure 14**

**Data-Driven Design Matrix**

|  | Morphometry | Anatomical Connectivity | Resting-State Functional Connectivity | Clinical Measures | Group |
| --- | --- | --- | --- | --- | --- |
| Morphometry | 0 | 0.831 | 0.678 | 0.560 | 1 |
| Anatomical Connectivity | 0.831 | 0 | 0.022 | 0.758 | 1 |
| Resting-State Functional Connectivity | 0.678 | 0.022 | 0 | 0.642 | 1 |
| Clinical Measures | 0.560 | 0.758 | 0.642 | 0 | 1 |
| Group | 1 | 1 | 1 | 1 | 0 |

Design matrix for DIABLO analysis for classifying nonobese and obese participants. Values are determined by taking the correlation between the first component from each dataset’s sPLS analysis.

**Supplementary Figure 15**

**Data-Driven Design Matrix**

|  | Morphometry | Anatomical Connectivity | Resting-State Functional Connectivity | Clinical Measures | Group |
| --- | --- | --- | --- | --- | --- |
| Morphometry | 0 | 0.916 | 0.832 | 0.778 | 1 |
| Anatomical Connectivity | 0.916 | 0 | 0.009 | 0.916 | 1 |
| Resting-State Functional Connectivity | 0.832 | 0.009 | 0 | 0.795 | 1 |
| Clinical Measures | 0.778 | 0.916 | 0.795 | 0 | 1 |
| Group | 1 | 1 | 1 | 1 | 0 |

Design matrix for DIABLO analysis for classifying obese male and obese female participants on the training set. Values are determined by taking the correlation between the first component from each dataset’s sPLS analysis.

**Supplementary Table 1: ROI Translations**

| *Abbreviation* | *Translation* |
| --- | --- |
| R | Right |
| L | Left |
| SA | Surface area |
| Vol | Volume |
| MC | Mean curvature |
| CT | Cortical thickness |

| *Shorthand* | *Morphological Abbr.* | *Full Name* |
| --- | --- | --- |
| CeB | Cerebellum Cortex | Cerebellar cortex |
| Tha | Thalamus Proper | Thalamus proper |
| CaN | Caudate | Caudate nucleus |
| Pu | Putamen | Putamen |
| Pal | Pallidum | Pallidum |
| Bstem | Brain Stem | Brain stem |
| Hip | Hippocampus | Hippocampus |
| Amg | Amygdala | Amygdala |
| Nacc | Accumbens area | (Nucleus) Accumbens area |
| FMarG S | G S frontomargin | Fronto-marginal gyrus (of Wernicke) and sulcus |
| InfOcG S | G S occipital inf | Inferior occipital gyrus (O3) and sulcus |
| PaCL S | G S paracentral | Paracentral lobule and sulcus |
| SbCG S | G S subcentral | Subcentral gyrus (central operculum) and sulci |
| TrFPoG S | G S transv frontopol | Transverse frontopolar gyri and sulci |
| ACgG S | G S cingul Ant | Anterior part of thecingulate gyrus and sulcus(ACC) |
| MACgG S | G S cingul Mid Ant | Middle-anterior part of the cingulate gyrus and sulcus (aMCC) |
| MPosCgG S | G S cingul Mid Post | Middle-posterior part of the cingulate gyrus and sulcus (pMCC) |
| PosDCgG | G cingul Post dorsal | Posterior-dorsal part of the cingulate gyrus (dPCC) |
| PosVCgG | G cingul Post ventral | Posterior-ventral part of the cingulate gyrus (vPCC, isthmus of the cingulate gyrus) |
| Cun | G cuneus | Cuneus (O6) |
| InfFGOpp | G front inf Opercular | Opercular part of the inferior frontal gyrus |
| InfFGOrp | G front inf Orbital | Orbital part of the inferior frontal gyrus |
| InfFGTrip | G front inf Triangul | Triangular part of the inferior frontal gyrus |
| MFG | G front middle | Middle frontal gyrus (F2) |
| SupFG | G front sup | Superior frontal gyrus (F1) |
| LoInG CInS | G Ins lg and S cent ins | Long insular gyrus and central sulcus of the insula |
| ShoInG | G insular short | Short insular gyri |
| MOcG | G occipital middle | Middle occipital gyrus (O2, lateral occipital gyrus) |
| SupOcG | G occipital sup | Superior occipital gyrus (O1) |
| FuG | G oc temp lat fusifor | Lateral occipito-temporal gyrus (fusiform gyrus, O4-T4) |
| LinG | G oc temp med Lingual | Lingual gyrus,ligual part of the medial occipito-temporal gyrus, (O5) |
| PaHipG | G oc temp med Parahip | Parahippocampal gyrus/parahippocampal part of the medial occipito-temporal gyrus (T5) |
| OrG | G orbital | Orbital gyri |
| AngG | G pariet inf Angular | Angular gyrus |
| SuMarG | G pariet inf Supramar | Supramarginal gyrus |
| SupPL | G parietal sup | Superior parietal lobule (lateral part of P1) |
| PosCG | G postcentral | Postcentral gyrus |
| PRCG | G precentral | Precentral gyrus |
| PrCun | G precuneus | Precuneus (medial part of P1) |
| RG | G rectus | Straight gyrus, Gyrus rectus |
| SbCaG | G subcallosal | Subcallosal area, subcallosal gyrus |
| HG | G temp sup G T transv | Anterior transverse temporal gyrus (of Heschl) |
| SupTGLp | G temp sup Lateral | Lateral aspect of the superior temporal gyrus |
| PoPl | G temp sup Plan polar | Planum polare of the superior temporal gyrus |
| TPl | G temp sup Plan tempo | Planum temporale or temporal plane of the superior temporal gyrus |
| InfTG | G temporal inf | Inferior temporal gyrus (T3) |
| MTG | G temporal middle | Middle temporal gyrus (T2) |
| ALSHorp | Lat Fis ant Horizont | Horizontal ramus of the anterior segment of the lateral sulcus (or fissure) |
| ALSVerp | Lat Fis ant Vertical | Vertical ramus of the anterior segment of the lateral sulcus (or fissure) |
| PosLS | Lat Fis post | Posterior ramus (or segment) of the lateral sulcus (or fissure) |
| OcPo | Pole occipital | Occipital pole |
| Tpo | Pole temporal | Temporal pole |
| CcS | S calcarine | Calcarine sulcus |
| CS | S central | Central sulcus (Rolando's fissure) |
| CgSMarp | S cingul Marginalis | Marginal branch (or part) of the cingulate sulcus |
| ACirIns | S circular insula ant | Anterior segment of the circular sulcus of the insula |
| InfCirIns | S circular insula inf | Inferior segment of the circular sulcus of the insula |
| SupCirInS | S circular insula sup | Superior segment of the circular sulcus of the insula |
| ATrCoS | S collat transv ant | Anterior transverse collateral sulcus |
| PosTrCoS | S collat transv post | Posterior transverse collateral sulcus |
| InfFS | S front inf | Inferior frontal sulcus |
| MFS | S front middle | Middle frontal sulcus |
| SupFS | S front sup | Superior frontal sulcus |
| JS | S interm prim Jensen | Sulcus intermedius primus (of Jensen) |
| IntPS TrPS | S intrapariet and P trans | Intraparietal sulcus (interparietal sulcus) and transverse parietal sulci |
| MOcS LuS | S oc middle and Lunatus | Middle occipital sulcus and lunatus sulcus |
| SupOcS TrOcS | S oc sup and transversal | Superior occipital sulcus and transverse occipital sulcus |
| AOcS | S occipital ant | Anterior occipital sulcus and preoccipital notch (temporo-occipital incisure) |
| LOcTS | S oc temp lat | Lateral occipito-temporal sulcus |
| CoS LinS | S oc temp med Lingual | Medial occipito-temporal sulcus (collateral sulcus) and lingual sulcus |
| LORs | S orbital lateral | Lateral orbital sulcus |
| MedOrS | S orbital med olfact | Medial orbital sulcus (olfactory sulcus) |
| OrS | S orbital H Shaped | Orbital sulci (H-shaped sulci) |
| POcS | S parieto occipital | Parieto-occipital sulcus (or fissure) |
| PerCaS | S pericallosal | Pericallosal sulcus (S of corpus callosum) |
| PosCS | S postcentral | Postcentral sulcus |
| InfPrCS | S precentral inf part | Inferior part of the precentral sulcus |
| SupPrCs | S precentral sup part | Superior part of the precentral sulcus |
| SbOrS | S suborbital | Suborbital sulcus (sulcus rostrales, supraorbital sulcus) |
| SbPS | S subparietal | Subparietal sulcus |
| InfTS | S temporal inf | Inferior temporal sulcus |
| SupTS | S temporal sup | Superior temporal sulcus (parallel sulcus) |
| TrTs | S temporal transverse | Transverse temporal sulcus |
| TPI | N/A | Planum temporale or temporal plane of the superior temporal gyrus |
| PosCgG S | N/A | Middle-posterior part of the cingulate gyrus and sulcus (pMCC) |
| N/A | x3rd Ventricle | Third Ventricle |
| N/A | Lateral Ventricle | Lateral Ventricle |
| N/A | Inf Lat Vent | Inferior horn of the lateral ventricle |
| N/A | Choroid plexus | Choroid plexus |

**Supplementary Table 2: Clinical Differences between High BMI and Normal BMI**

| **Characteristic** | **N** | **Nonobese BMI^1^**  N = 105 | **High  BMI^1^**  N = 78 | **p-value^2^** | **q-value^3^** | **Cohen’s D  (95% CI)^4^** |
| --- | --- | --- | --- | --- | --- | --- |
| **BRS Score** | 180 | 23.2 (4.2) | 22.8 (4.9) | 0.6 | 0.7 | 0.09 (-0.21, 0.38) |
| Unknown |  | 2 | 1 |  |  |  |
| **BSQ Abdominal Pain** | 53 | 1.36 (2.34) | 1.79 (2.82) | 0.6 | 0.7 | -0.15 (-0.82, 0.51) |
| Unknown |  | 94 | 36 |  |  |  |
| **BSQ Bloating** | 123 | 1.4 (2.7) | 4.2 (5.0) | 0.002 | 0.014 | -0.77 (-1.2, -0.38) |
| Unknown |  | 21 | 39 |  |  |  |
| **BSQ Overall Score** | 19 | 1.2 (1.6) | 4.9 (3.4) | 0.006 | 0.020 | -1.2 (-2.3, -0.09) |
| Unknown |  | 100 | 64 |  |  |  |
| **CDRISC Adaptability Score** | 182 | 17.02 (2.64) | 16.99 (2.76) | >0.9 | >0.9 | 0.01 (-0.28, 0.31) |
| Unknown |  | 1 | 0 |  |  |  |
| **CDRISC Control Meaning Score** | 182 | 9.52 (1.87) | 9.50 (1.95) | >0.9 | >0.9 | 0.01 (-0.28, 0.30) |
| Unknown |  | 1 | 0 |  |  |  |
| **CDRISC Emotional Cognitive Score** | 182 | 20.4 (4.2) | 20.7 (4.6) | 0.7 | 0.8 | -0.06 (-0.36, 0.23) |
| Unknown |  | 1 | 0 |  |  |  |
| **CDRISC Persistence Score** | 182 | 25.9 (4.5) | 26.0 (4.3) | 0.8 | 0.9 | -0.03 (-0.32, 0.26) |
| Unknown |  | 1 | 0 |  |  |  |
| **CDRISC Score** | 182 | 78 (12) | 79 (13) | 0.7 | 0.8 | -0.06 (-0.36, 0.23) |
| Unknown |  | 1 | 0 |  |  |  |
| **CTES Trauma Count** | 143 | 0.90 (0.91) | 1.26 (1.28) | 0.048 | 0.10 | -0.33 (-0.66, 0.00) |
| Unknown |  | 38 | 2 |  |  |  |
| **CTES Trauma Expression** | 92 | 2.56 (1.80) | 3.36 (1.77) | 0.034 | 0.078 | -0.45 (-0.86, -0.03) |
| Unknown |  | 62 | 29 |  |  |  |
| **CTES Trauma Overall** | 143 | 3.6 (4.6) | 6.3 (6.9) | 0.007 | 0.020 | -0.45 (-0.78, -0.12) |
| Unknown |  | 38 | 2 |  |  |  |
| **ETI Emotional Score** | 180 | 0.65 (1.36) | 1.35 (1.63) | 0.003 | 0.014 | -0.47 (-0.77, -0.17) |
| Unknown |  | 2 | 1 |  |  |  |
| **ETI General Score** | 181 | 1.29 (1.56) | 2.12 (2.11) | 0.004 | 0.019 | -0.46 (-0.75, -0.16) |
| Unknown |  | 1 | 1 |  |  |  |
| **ETI Physical Score** | 180 | 1.11 (1.40) | 1.44 (1.47) | 0.13 | 0.2 | -0.23 (-0.53, 0.06) |
| Unknown |  | 2 | 1 |  |  |  |
| **ETI Sexual Score** | 181 | 0.30 (0.81) | 0.86 (1.59) | 0.006 | 0.020 | -0.46 (-0.76, -0.17) |
| Unknown |  | 1 | 1 |  |  |  |
| **ETI Total Score** | 180 | 3.4 (3.6) | 5.8 (4.9) | <0.001 | 0.004 | -0.57 (-0.87, -0.27) |
| Unknown |  | 2 | 1 |  |  |  |
| **HAD Anxiety** | 183 | 3.5 (3.3) | 5.1 (3.5) | 0.002 | 0.013 | -0.48 (-0.77, -0.18) |
| **HAD Depression** | 183 | 1.65 (2.00) | 2.45 (2.68) | 0.028 | 0.072 | -0.35 (-0.64, -0.05) |
| **IPIP Extraversion** | 181 | 35 (6) | 36 (8) | 0.2 | 0.3 | -0.19 (-0.48, 0.11) |
| Unknown |  | 1 | 1 |  |  |  |
| **IPIP Neuroticism** | 181 | 21 (6) | 22 (7) | 0.2 | 0.3 | -0.20 (-0.50, 0.09) |
| Unknown |  | 1 | 1 |  |  |  |
| **PCS Helplessness** | 23 | 4.2 (6.0) | 3.6 (4.9) | 0.8 | 0.8 | 0.12 (-0.77, 1.0) |
| Unknown |  | 89 | 71 |  |  |  |
| **PCS Magnification** | 23 | 2.44 (2.66) | 1.14 (2.04) | 0.2 | 0.3 | 0.52 (-0.39, 1.4) |
| Unknown |  | 89 | 71 |  |  |  |
| **PCS Overall** | 23 | 10 (12) | 7 (10) | 0.6 | 0.7 | 0.24 (-0.65, 1.1) |
| Unknown |  | 89 | 71 |  |  |  |
| **PCS Rumination** | 23 | 3.31 (3.98) | 2.43 (3.51) | 0.6 | 0.7 | 0.23 (-0.66, 1.1) |
| Unknown |  | 89 | 71 |  |  |  |
| **Perceived Stress Scale Score** | 181 | 11 (6) | 12 (6) | 0.3 | 0.4 | -0.17 (-0.46, 0.13) |
| Unknown |  | 0 | 2 |  |  |  |
| **PILL Score** | 176 | 5.1 (4.5) | 8.7 (6.6) | <0.001 | 0.002 | -0.65 (-1.0, -0.34) |
| Unknown |  | 6 | 1 |  |  |  |
| **PROMIS Sleep Score** | 179 | 45 (8) | 45 (9) | 0.6 | 0.7 | -0.09 (-0.39, 0.20) |
| Unknown |  | 4 | 0 |  |  |  |
| **SF12 Mental Component Score** | 183 | 53 (6) | 50 (9) | 0.017 | 0.045 | 0.39 (0.09, 0.68) |
| **SF12 Physical Component Score** | 183 | 55.0 (3.1) | 53.4 (4.3) | 0.007 | 0.020 | 0.43 (0.13, 0.73) |
| **STAI Trait Anxiety** | 181 | 46 (9) | 48 (11) | 0.2 | 0.3 | -0.19 (-0.48, 0.11) |
| Unknown |  | 1 | 1 |  |  |  |
| **VSI Score** | 182 | 5 (9) | 8 (10) | 0.030 | 0.073 | -0.33 (-0.63, -0.03) |
| Unknown |  | 1 | 0 |  |  |  |
| **YFAS Clinically Significant Impairment** | 168 | 0.08 (0.37) | 0.12 (0.40) | 0.5 | 0.7 | -0.11 (-0.41, 0.19) |
| Unknown |  | 13 | 2 |  |  |  |
| **YFAS Continued Use** | 168 | 0.09 (0.28) | 0.28 (0.45) | 0.002 | 0.013 | -0.51 (-0.82, -0.20) |
| Unknown |  | 13 | 2 |  |  |  |
| **YFAS Given Up** | 168 | 0.11 (0.43) | 0.21 (0.72) | 0.3 | 0.4 | -0.18 (-0.48, 0.13) |
| Unknown |  | 13 | 2 |  |  |  |
| **YFAS Loss of Control** | 167 | 0.05 (0.31) | 0.13 (0.41) | 0.2 | 0.3 | -0.22 (-0.52, 0.09) |
| Unknown |  | 13 | 3 |  |  |  |
| **YFAS Symptom Count** | 168 | 1.24 (1.10) | 2.07 (1.53) | <0.001 | 0.002 | -0.63 (-0.94, -0.32) |
| Unknown |  | 13 | 2 |  |  |  |
| **YFAS Time Spent** | 168 | 0.11 (0.40) | 0.34 (0.60) | 0.005 | 0.019 | -0.46 (-0.77, -0.16) |
| Unknown |  | 13 | 2 |  |  |  |
| **YFAS Tolerance** | 163 | 0.09 (0.35) | 0.24 (0.57) | 0.056 | 0.11 | -0.32 (-0.63, -0.01) |
| Unknown |  | 14 | 6 |  |  |  |
| **YFAS Unsuccessful Cutdown** | 157 | 1.30 (0.71) | 1.82 (0.95) | <0.001 | 0.002 | -0.63 (-1.0, -0.31) |
| Unknown |  | 21 | 5 |  |  |  |
| **YFAS Withdrawal** | 168 | 0.12 (0.49) | 0.25 (0.57) | 0.12 | 0.2 | -0.25 (-0.55, 0.06) |
| Unknown |  | 13 | 2 |  |  |  |
| ^1^Mean (SD); n / N (%) | | | | | | |
| ^2^Welch Two Sample t-test | | | | | | |
| ^3^False discovery rate correction for multiple testing | | | | | | |
| ^4^Cohen's D (95% CI) | | | | | | |

**Supplementary Table 3: Clinical Differences between High Males BMI and High Females BMI**

| **Characteristic** | **N** | **High BMI Male^1^**  N = 23 | **High BMI Female^1^**  N = 55 | **p-value^2^** | **q-value^3^** | **Cohen’s D**  **(95% CI)^4^** |
| --- | --- | --- | --- | --- | --- | --- |
| **BRS Score** | 77 | 23.8 (3.5) | 22.4 (5.4) | 0.2 | 0.7 | 0.27 (-0.22, 0.76) |
| Unknown |  | 0 | 1 |  |  |  |
| **CDRISC Adaptability Score** | 78 | 17.61 (2.39) | 16.73 (2.88) | 0.2 | 0.7 | 0.32 (-0.17, 0.81) |
| **CDRISC Control/Meaning Score** | 78 | 9.30 (2.30) | 9.58 (1.79) | 0.6 | 0.8 | -0.14 (-0.63, 0.35) |
| **CDRISC Emotional Cognitive Score** | 78 | 21.2 (4.1) | 20.5 (4.8) | 0.5 | 0.8 | 0.14 (-0.34, 0.63) |
| **CDRISC Persistence Score** | 78 | 26.9 (3.4) | 25.7 (4.7) | 0.2 | 0.7 | 0.27 (-0.22, 0.76) |
| **CDRISC Total Score** | 78 | 81 (12) | 78 (14) | 0.4 | 0.7 | 0.22 (-0.27, 0.71) |
| **CTES Trauma Count** | 76 | 1.05 (1.02) | 1.35 (1.36) | 0.3 | 0.7 | -0.23 (-0.74, 0.27) |
| Unknown |  | 2 | 0 |  |  |  |
| **CTES Trauma Expression** | 49 | 3.54 (1.83) | 3.30 (1.77) | 0.7 | 0.8 | 0.14 (-0.50, 0.77) |
| Unknown |  | 10 | 19 |  |  |  |
| **CTES Trauma Overall** | 76 | 5.2 (5.3) | 6.7 (7.4) | 0.3 | 0.7 | -0.22 (-0.73, 0.28) |
| Unknown |  | 2 | 0 |  |  |  |
| **ETI Emotional Score** | 77 | 1.18 (1.62) | 1.42 (1.64) | 0.6 | 0.8 | -0.14 (-0.64, 0.35) |
| Unknown |  | 1 | 0 |  |  |  |
| **ETI Physical Score** | 77 | 1.64 (1.65) | 1.36 (1.41) | 0.5 | 0.8 | 0.18 (-0.31, 0.68) |
| Unknown |  | 1 | 0 |  |  |  |
| **ETI Total Score** | 77 | 5.7 (5.2) | 5.8 (4.9) | >0.9 | >0.9 | -0.02 (-0.52, 0.47) |
| Unknown |  | 1 | 0 |  |  |  |
| **HAD Anxiety** | 78 | 4.2 (2.5) | 5.4 (3.8) | 0.11 | 0.7 | -0.34 (-0.83, 0.15) |
| **HAD Depression** | 78 | 1.96 (2.57) | 2.65 (2.72) | 0.3 | 0.7 | -0.26 (-0.75, 0.23) |
| **IPIP Extraversion** | 77 | 38 (7) | 35 (8) | 0.2 | 0.7 | 0.34 (-0.15, 0.83) |
| Unknown |  | 0 | 1 |  |  |  |
| **IPIP Neuroticism** | 77 | 20 (5) | 23 (8) | 0.039 | 0.7 | -0.44 (-0.93, 0.05) |
| Unknown |  | 0 | 1 |  |  |  |
| **Percieved Stress Score** | 76 | 11.4 (6.5) | 12.9 (6.3) | 0.4 | 0.7 | -0.23 (-0.72, 0.26) |
| Unknown |  | 0 | 2 |  |  |  |
| **PILL Score** | 77 | 9 (7) | 9 (7) | >0.9 | >0.9 | -0.03 (-0.52, 0.46) |
| Unknown |  | 1 | 0 |  |  |  |
| **PROMIS Sleep Score** | 78 | 44 (9) | 46 (9) | 0.4 | 0.8 | -0.20 (-0.68, 0.29) |
| **SF12 Mental Component Score** | 78 | 50 (10) | 51 (9) | 0.7 | 0.8 | -0.10 (-0.58, 0.39) |
| **SF12 Physical Component Score** | 78 | 54.2 (4.6) | 53.1 (4.2) | 0.3 | 0.7 | 0.27 (-0.22, 0.76) |
| **Trait Anxiety** | 77 | 48 (9) | 48 (12) | >0.9 | >0.9 | 0.01 (-0.48, 0.49) |
| Unknown |  | 0 | 1 |  |  |  |
| **VSI Score** | 78 | 8 (8) | 9 (11) | 0.6 | 0.8 | -0.10 (-0.59, 0.39) |
| **YFAS Continued Use** | 76 | 0.17 (0.39) | 0.32 (0.47) | 0.2 | 0.7 | -0.33 (-0.82, 0.17) |
| Unknown |  | 0 | 2 |  |  |  |
| **YFAS Symptom Count** | 76 | 1.91 (1.56) | 2.13 (1.52) | 0.6 | 0.8 | -0.14 (-0.63, 0.35) |
| Unknown |  | 0 | 2 |  |  |  |
| **YFAS Time Spent** | 76 | 0.26 (0.54) | 0.38 (0.63) | 0.4 | 0.8 | -0.19 (-0.68, 0.30) |
| Unknown |  | 0 | 2 |  |  |  |
| **YFAS Unsuccessful Cutdown** | 73 | 1.91 (1.15) | 1.78 (0.86) | 0.7 | 0.8 | 0.13 (-0.37, 0.63) |
| Unknown |  | 1 | 4 |  |  |  |
| ^1^Mean (SD); n / N (%) | | | | | | |
| ^2^Welch Two Sample t-test | | | | | | |
| ^3^False discovery rate correction for multiple testing | | | | | | |
| ^4^Cohen's D (95% CI) | | | | | | |

**Supplementary Table 4: Nonobese vs Obese**

| **Block** | **Component** | **AUC** | **p-value** |
| --- | --- | --- | --- |
| *Morphometry* | 1 | 0.540 | 0.435 |
|  | 2 | 0.622 | 0.018 |
| *Anatomical Connectivity* | 1 | 0.525 | 0.627 |
|  | 2 | 0.572 | 0.164 |
| *Resting-State Functional Connectivity* | 1 | 0.704 | 7.88e-05 |
|  | 2 | 0.773 | 1.16e-07 |
| *Behavioral/Clinical* | 1 | 0.579 | 0.122 |
|  | 2 | 0.719 | 2.18e-05 |
| *Combined* | 1 | 0.763 | 0.0019 |
|  | 2 | 0.810 | 2.85e-06 |

Area under the curve (AUC) for the receiving operating characteristic per block and component, along with the full combined DIABLO model predicting nonobese participants from obese participants. The p-value results from a Wilcoxon test comparing the two classes.

**Supplementary Table 5: Obese Male and Obese Female**

| **Block** | **Component** | **AUC** | **p-value** |
| --- | --- | --- | --- |
| *Morphometry* | 1 | 0.634 | 0.183 |
|  | 2 | 0.813 | 0.0002 |
| *Anatomical Connectivity* | 1 | 0.727 | 0.023 |
|  | 2 | 0.783 | 0.005 |
| *Resting-State Functional Connectivity* | 1 | 0.674 | 0.083 |
|  | 2 | 0.841 | 0.0007 |
| *Behavioral/Clinical* | 1 | 0.568 | 0.498 |
|  | 2 | 0.770 | 0.007 |
| *Combined* | 1 | 0.815 | 0.023 |
|  | 2 | 0.908 | 0.0018 |

Area under the curve (AUC) for the receiving operating characteristic per block and component, along with the full combined DIABLO model predicting obese male participants from obese female participants. The p-value results from a Wilcoxon test comparing the two classes.

**Supplementary References**

Baik, S.H., et al., 2019. Reliability and validity of the Perceived Stress Scale-10 in Hispanic Americans with English or Spanish language preference. Journal of health psychology. 24**,** 628-639.

Bremner, J.D., Vermetten, E., Mazure, C.M., 2000. Development and preliminary psychometric properties of an instrument for the measurement of childhood trauma: the Early Trauma Inventory. Depress Anxiety. 12**,** 1-12.

Bremner, J.D., Bolus, R., Mayer, E.A., 2007. Psychometric properties of the Early Trauma Inventory-Self Report. The Journal of nervous and mental disease. 195**,** 211-218.

Chmitorz, A., et al., 2018. Population-based validation of a German version of the Brief Resilience Scale. PLOS ONE. 13**,** e0192761.

Connor, K.M., Davidson, J.R.T., 2003. Development of a new resilience scale: The Connor-Davidson Resilience Scale (CD-RISC). Depression and Anxiety. 18**,** 76-82.

Fischl, B., 2012. FreeSurfer. Neuroimage. 62**,** 774-81.

Fountoulakis, K., et al., 2006. Annals of General Psychiatry. 5**,** 2.

Frazier, J.A., et al., 2005. Structural Brain Magnetic Resonance Imaging of Limbic and Thalamic Volumes in Pediatric Bipolar Disorder. American Journal of Psychiatry. 162**,** 1256-1265.

Fung, S.-F., 2020. Validity of the Brief Resilience Scale and Brief Resilient Coping Scale in a Chinese Sample. International journal of environmental research and public health. 17**,** 1265.

Gearhardt, A.N., Corbin, W.R., Brownell, K.D., 2009. Preliminary validation of the Yale Food Addiction Scale. Appetite. 52**,** 430-6.

Gearhardt, A.N., et al., 2012. An examination of the food addiction construct in obese patients with binge eating disorder. Int J Eat Disord. 45**,** 657-63.

Gibbons, D., et al., 2020. Advantages and Design of PROMIS Questionnaires. Clin Spine Surg. 33**,** 408-410.

Glasser, M.F., et al., 2016. A multi-modal parcellation of human cerebral cortex. Nature. 536**,** 171-178.

Goldberg, L.R., et al., 2006. The international personality item pool and the future of public-domain personality measures. Journal of Research in Personality. 40**,** 84-96.

Goldstein, J.M., et al., 2007. Hypothalamic abnormalities in schizophrenia: sex effects and genetic vulnerability. Biol Psychiatry. 61**,** 935-45.

Gupta, A., et al., 2017. Sex differences in the influence of body mass index on anatomical architecture of brain networks. International Journal of Obesity. 41**,** 1185-1195.

Gustafson, L.W., et al., 2020. Validity and reliability of State-Trait Anxiety Inventory in Danish women aged 45 years and older with abnormal cervical screening results. BMC Med Res Methodol. 20**,** 89.

Huo, T., et al., 2018. Assessing the reliability of the short form 12 (SF-12) health survey in adults with mental health conditions: a report from the wellness incentive and navigation (WIN) study. Health and quality of life outcomes. 16**,** 34-34.

Iwata, N., et al., 1998. The Japanese adaptation of the STAI Form Y in Japanese working adults--the presence or absence of anxiety. Ind Health. 36**,** 8-13.

Julian, L.J., 2011. Measures of anxiety: State-Trait Anxiety Inventory (STAI), Beck Anxiety Inventory (BAI), and Hospital Anxiety and Depression Scale-Anxiety (HADS-A). Arthritis Care & Research. 63**,** S467-S472.

Kunzler, A.M., et al., 2018. Construct Validity and Population-Based Norms of the German Brief Resilience Scale (BRS). European journal of health psychology. 25**,** 107-117.

Labus, J.S., et al., 2004. The Visceral Sensitivity Index: development and validation of a gastrointestinal symptom-specific anxiety scale. Aliment Pharmacol Ther. 20**,** 89-97.

Larson, C.O., 2002. Use of the SF-12 instrument for measuring the health of homeless persons. Health services research. 37**,** 733-750.

Lee, R.L., Mok, E.S., 2011. Evaluation of the psychometric properties of a modified Chinese version of the Caregiver Task Inventory--refinement and psychometric testing of the Chinese Caregiver Task Inventory: a confirmatory factor analysis. J Clin Nurs. 20**,** 3452-62.

Makris, N., et al., 2006. Decreased volume of left and total anterior insular lobule in schizophrenia. Schizophr Res. 83**,** 155-71.

Nativ-Zeltzer, N., et al., 2019. Validation of the PILL-5: A 5-Item Patient Reported Outcome Measure for Pill Dysphagia. Frontiers in surgery. 6**,** 43-43.

Park, S.H., et al., 2018. Resilience is decreased in irritable bowel syndrome and associated with symptoms and cortisol response. Neurogastroenterology & Motility. 30**,** e13155.

Pletikosic Toncic, S., Tkalcic, M., 2017. A Measure of Suffering in relation to Anxiety and Quality of Life in IBS Patients: Preliminary Results. Biomed Res Int. 2017**,** 2387681.

Quek, K.F., et al., 2004. Reliability and validity of the Spielberger State-Trait Anxiety Inventory (STAI) among urological patients: a Malaysian study. Med J Malaysia. 59**,** 258-67.

Rishi, P., et al., 2017. Hospital anxiety and depression scale assessment of 100 patients before and after using low vision care: A prospective study in a tertiary eye-care setting. Indian journal of ophthalmology. 65**,** 1203-1208.

Talley, N.J., et al., 1995. Initial validation of a bowel symptom questionnaire* and measurement of chronic gastrointestinal symptoms in Australians. Australian and New Zealand Journal of Medicine. 25**,** 302-308.

Tansey, T.N., et al., 2015. Psychometric Validation of the Brief Resilience Scale in a Sample of Vocational Rehabilitation Consumers. Rehabilitation Counseling Bulletin. 59**,** 108-111.

Walvekar, S.S., Ambekar, J.G., Devaranavadagi, B.B., 2015. Study on serum cortisol and perceived stress scale in the police constables. Journal of clinical and diagnostic research : JCDR. 9**,** BC10-BC14.

Zigmond, A.S., Snaith, R.P., 1983. The hospital anxiety and depression scale. Acta Psychiatr Scand. 67**,** 361-70.
